# Nationwide multicenter questionnaire surveys on countermeasures against antimicrobial resistance and infections in hospitals

**DOI:** 10.1186/s12879-021-05921-2

**Published:** 2021-02-27

**Authors:** Jung-ho Shin, Seiko Mizuno, Takuya Okuno, Hisashi Itoshima, Noriko Sasaki, Susumu Kunisawa, Mitsuo Kaku, Makiko Yoshida, Yoshiaki Gu, Daiichi Morii, Keigo Shibayama, Norio Ohmagari, Yuichi Imanaka

**Affiliations:** 1 Department of Healthcare Economics and Quality Management, Graduate School of Medicine, Kyoto University, Yoshida Konoe-cho, Sakyo-ku, Kyoto, 606-8501 Japan; 2 Division of Infectious Diseases and Infection Control, Faculty of Medicine, Tohoku Medical and Pharmaceutical University, Sendai, Japan; 3 Department of Infectious Diseases, Tohoku University Graduate School of Medicine, Sendai, Japan; 4 AMR Clinical Reference Center, National Center for Global Health and Medicine Hospital, Tokyo, Japan; 5 Department of Infection Control and Prevention, Graduate School of Medicine, Osaka University, Suita, Osaka, Japan; 6 Department of Bacteriology II, National Institute of Infectious Diseases, Tokyo, Japan; 7 Department of Infectious Diseases, AMR Clinical Reference Center, and Disease Control and Prevention Center, National Center for Global Health and Medicine Hospital, Tokyo, Japan

**Keywords:** Antimicrobial resistance, Antimicrobial stewardship, Healthcare-associated infection, Infection control, Surveillance

## Abstract

**Background:**

The goals of the National Action Plan on Antimicrobial Resistance (AMR) of Japan include “implementing appropriate infection prevention and control” and “appropriate use of antimicrobials,” which are relevant to healthcare facilities. Specifically, linking efforts between existing infection control teams and antimicrobial stewardship programs was suggested to be important. Previous studies reported that human resources, such as full-time equivalents of infection control practitioners, were related to improvements in antimicrobial stewardship.

**Methods:**

We posted questionnaires to all teaching hospitals (*n* = 1017) regarding hospital countermeasures against AMR and infections. To evaluate changes over time, surveys were conducted twice (1st survey: Nov 2016, 2nd survey: Feb 2018). A latent transition analysis (LTA) was performed to identify latent statuses, which refer to underlying subgroups of hospitals, and effects of the number of members in infection control teams per bed on being in the better statuses.

**Results:**

The number of valid responses was 678 (response rate, 66.7%) for the 1st survey and 559 (55.0%) for the 2nd survey. More than 99% of participating hospitals had infection control teams, with differences in activity among hospitals. Roughly 70% had their own intervention criteria for antibiotics therapies, whereas only about 60 and 50% had criteria established for the use of anti-methicillin-resistant *Staphylococcus aureus* antibiotics and broad-spectrum antibiotics, respectively. Only 50 and 40% of hospitals conducted surveillance of catheter-associated urinary tract infections and ventilator-associated pneumonia, respectively. Less than 50% of hospitals used maximal barrier precautions for central line catheter insertion.

The LTA identified five latent statuses. The membership probability of the most favorable status in the 2nd study period was slightly increased from the 1st study period (23.6 to 25.3%). However, the increase in the least favorable status was higher (26.3 to 31.8%). Results of the LTA did not support a relationship between increasing the number of infection control practitioners per bed, which is reportedly related to improvements in antimicrobial stewardship, and being in more favorable latent statuses.

**Conclusions:**

Our results suggest the need for more comprehensive antimicrobial stewardship programs and increased surveillance activities for healthcare-associated infections to improve antimicrobial stewardship and infection control in hospitals.

**Supplementary Information:**

The online version contains supplementary material available at 10.1186/s12879-021-05921-2.

## Introduction

The World Health Assembly adopted the Global Action Plan on Antimicrobial Resistance (AMR) in May 2015 [1]. In Japan, the National Action Plan on Antimicrobial Resistance was adopted in April 2016 and included two goals [2], “implementing appropriate infection prevention and control” and “appropriate use of antimicrobials.” These goals are of particular relevance to healthcare facilities in terms of preventing the spread of antimicrobial-resistant organisms. Specifically, at the field level, linking efforts between existing infection control team (ICT) and antimicrobial stewardship (AMS) programs was suggested to be important [2].

Previous studies reported that human resources (expressed as full-time equivalents (FTEs) of infection control practitioners) and FTE-to-bed ratios were related to improvements in AMS [3–5]. However, the definition of improvement varied from study to study. For example, one study used an increase in the number of implemented AMS programs [3] to evaluate the performance of AMS, while another study examined the effectiveness of each program [4].

The purpose of the present study was two-fold: to report the results of nationwide multicenter questionnaire surveys on countermeasures against AMR and infections in Japanese teaching hospitals, and to identify latent statuses, which might imply underlying subgroups of hospitals with similar achievement levels of AMS, and examine the effects of FTE-to-bed ratios of ICT members on the latent statuses.

## Methods

We posted questionnaires to all teaching hospitals in Japan (*n* = 1017 as of 2015). To examine changes over a period of roughly 1 year, surveys were conducted twice in November 2016 and February 2018 (see the English translation of the questionnaires in Additional file 1). No intervention was provided by our study team between the two surveys. The contents of the questionnaire included basic information, such as the number of beds (1st survey only), questions divided into sections 1 to 12 for countermeasures against AMR based from a previous study [6] and a guide published by the Japanese Ministry of Health, Labour and Welfare [7], and section 13 for results of bacterial cultures, as follows: 1. Organizational structure for nosocomial infection control; 2. Activities of ICT; 3. Preventive measures by the route of infections; 4. Maintenance of medical equipment; 5. Standard precautions; 6. Ward; 7. Intensive care unit (ICU); 8. Operating room; 9. Prevention of postoperative infections; 10. Management of food hygiene in hospitals; 11. Management of medical waste; 12. Cleaning, disinfection, and sterilization of instruments; and 13. Antimicrobial-resistant organisms. The questions were answered (1) numerically (e.g., number of physicians) or by choosing (2) either “yes” or “no” or (3) one among three to five options in order (e.g., “in approximately 100%/80%/50%/20%/0% of relevant cases”).

We analyzed valid responses, which included hospital information to link the 1st and 2nd surveys. We excluded duplicate responses to the same survey by the same hospital. Answers to questions in sections 1 to 12 are presented as medians and interquartile ranges, calculated after excluding missing values. For single-choice questions, we presented the proportions of “yes” or the most favorable option (e.g., “approximately 100% of relevant cases”). For these types of questions, we created a “missing” category. Student’s t-tests or Satterthwaite tests were used for continuous variables, and Cochran-Mantel-Haenszel tests for categorical variables, to compare results from the 1st and 2nd surveys.

The answers to questions in section 13 were the results of surveillance in 2015 for the 1st survey and 2016 for the 2nd survey, which were 1 year before each survey. We calculated the proportions of isolated microorganisms and antimicrobial-resistant organisms for each hospital that responded to both surveys. Wilcoxon signed-rank tests were used, assuming that the results from the 1st and 2nd surveys regarding section 13 were paired data.

To study the achievement level for AMS programs of hospitals, we performed a latent transition analysis (LTA), which is a longitudinal extension of latent class analysis [8]. Latent class analysis identifies underlying subgroups in a population, but the characteristics of these underlying subgroups are hard to observe directly; these are indicated by several observed variables [8]. While latent class analysis identifies underlying (unobservable) subgroups within a population as “classes,” LTA refers to the subgroups as “statuses” to reflect the fact that membership in the subgroups can change over time [8]. In this study, we performed LTA using data from hospitals that responded to both surveys, and time periods 1 and 2 for LTA were defined as those of 1st and 2nd surveys, respectively. Questions for which the proportion of the most favorable answer was less than 80% in the 2nd survey were used to classify hospitals into subgroups, latent statuses, with similar sets of answers to these questions. We excluded questions regarding handwashing sinks in ICUs, for which the proportion of the most favorable answer was less than 80%, given the lack of established guidelines. We also reduced the multiple categories in each question to two (most favorable/others) to improve the precision of estimates [9]. FTEs of ICT members were selected as a covariate that might affect the membership probabilities for time period 1. We determined the number of statuses by considering interpretability and fit statistics, and presented the fit statistics, status membership probabilities, transition probabilities, item-response probabilities, and estimated odds ratios for covariates. The domains, which consisted of several questions, were determined empirically according to the LTA results.

SAS® software version 9.4 (SAS Institute Inc., Cary, NC, USA) was used for all analyses, and PROC LTA (version 1.3.2) was used for the LTA [10]. A two-tailed significance level of 0.05 was used for all tests.

## Results

Among 1017 teaching hospitals, 683 and 563 hospitals responded to the 1st and 2nd surveys, respectively. The numbers of valid responses were 678 for the 1st survey (response rate: 66.7%) and 559 for the 2nd survey (response rate: 55.0%) after excluding duplicated responses and those with missing hospital information. The number of hospitals that responded to both surveys was 437 (response rate: 43.0%).

The mean number of hospital beds was 434 (median, 389: 675 responses). Table 1 presents the results of the two surveys for all hospitals with valid responses and hospitals that responded to both surveys (see Tables S1 and S2 in Additional file 2 for more details). More than 99% of hospitals reported having active ICTs, with a median of 10 to 11 ICT members. Both crude numbers and FTEs of ICT members did not differ significantly between the 1st and 2nd surveys.

**Table 1:** Results of the 1st and 2nd questionnaire surveys

	All hospitals with valid responses					Hospitals that responded to both surveys				
Question	1st survey (*n* = 678)		2nd survey (*n* = 559)		P*	1st survey (*n* = 437)		2nd survey (*n* = 437)		P*
Number of staff										
Physician (full-time)	75	(47–128)	80	(48.5–137.5)	0.237	80	(50–140)	81	(50–137)	0.805
Nurse (full-time)	336	(235–528.5)	360	(251–561)	0.066	368	(246–543)	371	(251–561)	0.629
Laboratory technologist (full-time)	23	(16–34)	24	(17–36)	0.107	24	(17–36.5)	24.5	(17–37)	0.819
Pharmacist (full-time)	19	(13–28)	20	(14–30)	0.066	19	(14–28)	20	(14–30)	0.360
Dietitian	5	(4–8)	5	(4–8)	0.097	5	(4–8)	6	(4–8)	0.370
Administrative staff	52	(32–86)	53.5	(32–87)	0.611	56	(33–87)	56	(33–89)	0.718
Registered ICD (MD or PhD)	2	(1–4)	3	(2–4)	0.139	3	(2–4)	3	(2–4)	0.322
We have an active ICT.	674	(99.4%)	557	(99.6%)	0.843	436	(99.8%)	435	(99.5%)	0.607
Number of ICT member, crude	10	(8–16)	11	(7–16)	0.103	11	(8–17)	11	(7–17)	0.530
Physician	2.5	(2–4)	3	(2–4)	0.153	3	(2–4)	3	(2–4)	0.576
Nurse	2	(2–4)	2	(2–4)	0.488	2	(2–4)	2	(2–4)	0.757
Pharmacist	2	(1–2)	2	(1–2)	0.255	2	(1–2)	2	(1–2)	0.242
Laboratory technologist	2	(1–2)	2	(1–2)	0.230	2	(1–2)	2	(1–2)	0.709
Dietitian	0	(0–0)	0	(0–0)	0.910	0	(0–0)	0	(0–0)	0.948
Administrative staff	1	(0–2)	1	(0–1)	0.969	1	(0–2)	1	(1–2)	0.926
Number of ICT member, full-time equivalent	2.8	(1.3–4.3)	2.8	(1.8–4)	0.717	2.8	(1.6–4.3)	2.8	(1.8–4.1)	0.920
Physician	2.5	(2–4)	3	(2–4)	0.951	3	(2–4)	3	(2–4)	0.830
Nurse	0.8	(0.8–1.3)	0.8	(0.8–1.3)	0.675	0.8	(0.8–1.3)	0.8	(0.8–1.3)	0.693
Pharmacist	0.5	(0–0.8)	0.5	(0–0.8)	0.725	0.5	(0–0.8)	0.5	(0–0.65)	0.531
Laboratory technologist	0.5	(0–0.8)	0.5	(0–0.5)	0.953	0.5	(0–1)	0.5	(0–0.8)	0.931
Dietitian	0	(0–0)	0	(0–0)	0.068	0	(0–0)	0	(0–0)	0.067
Administrative staff	0	(0–0.5)	0	(0–0.5)	0.839	0	(0–0.5)	0	(0–0.5)	0.524
FTE per 100 beds	0.7	(0.4–1.0)	0.7	(0.4–1.0)	0.918	0.7	(0.4–1.0)	0.7	(0.4–1.0)	0.918
We performed bacterial culture, identification, and susceptibility tests basically in our hospital.	542	(79.9%)	466	(83.4%)	0.301	355	(81.2%)	367	(84.0%)	0.362
We participate in JANIS programs.	647	(95.4%)	548	(98.0%)	**0.025**	426	(97.5%)	432	(98.9%)	0.219
Clinical laboratory division	636	(93.8%)	536	(95.9%)	0.103	421	(96.3%)	422	(96.6%)	0.855
Antimicrobial-resistant bacterial infection division	311	(45.9%)	288	(51.5%)	**0.048**	228	(52.2%)	235	(53.8%)	0.635
Surgical site infection division	366	(54.0%)	324	(58.0%)	0.161	249	(57.0%)	259	(59.3%)	0.493
Intensive care unit division	116	(17.1%)	88	(15.7%)	0.519	80	(18.3%)	74	(16.9%)	0.595
Neonatal intensive care unit division	74	(10.9%)	64	(11.4%)	0.766	56	(12.8%)	51	(11.7%)	0.606
**1. Organizational structure for nosocomial infection control**										
The head of our hospital attends ICC almost every time.	576	(85.0%)	473	(84.6%)	**0.027**	379	(86.7%)	369	(84.4%)	**0.018**
We have a comprehensive hospital infection control manual that can be used all around our hospital.	677	(99.9%)	559	(100.0%)	0.364	437	(100.0%)	437	(100.0%)	–
We hold a workshop regarding countermeasures against hospital infection more than once a year.	677	(99.9%)	559	(100.0%)	0.364	437	(100.0%)	437	(100.0%)	–
We have tools, such as the intranet and bulletin boards, to inform our staff of hospital infection-related matters.	671	(99.0%)	556	(99.5%)	0.397	434	(99.3%)	436	(99.8%)	0.317
**2. Activities of ICT**										
We hold a regular ICT meeting.	628	(92.6%)	534	(95.5%)	**0.042**	410	(93.8%)	416	(95.2%)	0.353
We provide consultation as an activity of the ICT.	633	(93.4%)	516	(92.3%)	0.274	412	(94.3%)	407	(93.1%)	0.333
**We have an AST (a member can work for both ICT and AST).**	542	(79.9%)	373	(66.7%)	**<.001**	355	(81.2%)	305	(69.8%)	**<.001**
We monitor the uses of antibiotics to assure their propriety.	652	(96.2%)	544	(97.3%)	0.476	420	(96.1%)	431	(98.6%)	0.064
We intervene to assure appropriate uses of antibiotics.	631	(93.1%)	527	(94.3%)	0.177	410	(93.8%)	415	(95.0%)	0.317
**We have established criteria of interventions, such as their administration duration and selection, for patients administered antibiotics.**	466	(68.7%)	399	(71.4%)	0.589	304	(69.6%)	310	(70.9%)	0.691
**We have criteria for the uses of anti-MRSA antibiotics.**	433	(63.9%)	361	(64.6%)	0.964	267	(61.1%)	278	(63.6%)	0.594
We record the used amount of anti-MRSA antibiotics.	667	(98.4%)	554	(99.1%)	0.508	432	(98.9%)	432	(98.9%)	0.788
We have a reporting system (1st survey: “registration system”) for the use of anti-MRSA antibiotics.	390	(57.5%)	542	(97.0%)	**<.001**	259	(59.3%)	425	(97.3%)	**<.001**
**We have a preauthorization and/or restriction system for the use of anti-MRSA antibiotics.**	321	(47.3%)	208	(37.2%)	**<.001**	206	(47.1%)	169	(38.7%)	**0.035**
**We have criteria for the uses of broad-spectrum antibiotics such as carbapenems.**	355	(52.4%)	287	(51.3%)	0.369	217	(49.7%)	224	(51.3%)	0.305
We have a reporting system (1st survey: “registration system”) for the use of broad-spectrum antibiotics.	391	(57.7%)	530	(94.8%)	**<.001**	251	(57.4%)	415	(95.0%)	**<.001**
**We have a preauthorization and/or restriction system for the use of broad-spectrum antibiotics.**	258	(38.1%)	131	(23.4%)	**<.001**	157	(35.9%)	111	(25.4%)	**0.003**
We record the used amount of broad-spectrum antibiotics.	667	(98.4%)	550	(98.4%)	0.935	429	(98.2%)	431	(98.6%)	0.777
We have a reference system, such as the intranet of a booklet, for the antibiogram.	562	(82.9%)	482	(86.2%)	0.238	371	(84.9%)	383	(87.6%)	0.499
**We performed TDM for basically all cases.**	423	(62.4%)	362	(64.8%)	0.273	273	(62.5%)	287	(65.7%)	0.193
We record the vaccination proportion of employees who are HBsAg-negative.	581	(85.7%)	485	(86.8%)	0.415	369	(84.4%)	378	(86.5%)	0.096
We perform IGRAs for employees who are in contact with tuberculosis patients.	616	(90.9%)	503	(90.0%)	0.772	404	(92.4%)	397	(90.8%)	0.556
**We record employees’ immunization statuses for measles, rubella, chickenpox, and mumps (2nd survey: “for all of measles, rubella, chickenpox, and mumps”).**	572	(84.4%)	340	(60.8%)	**<.001**	371	(84.9%)	273	(62.5%)	**<.001**
We have a manual and a reporting system of needle punctures and sharp object injuries.	678	(100.0%)	559	(100.0%)	–	437	(100.0%)	437	(100.0%)	–
**Needle puncture and sharp object injuries are reported to a relevant department, such as ICT.**	463	(68.3%)	391	(69.9%)	0.177	301	(68.9%)	307	(70.3%)	0.408
**ICT and/or ICPs check the number of isolated antimicrobial-resistant organisms and other microorganisms that are relevant to infection control on a daily basis**	436	(64.3%)	357	(63.9%)	0.409	281	(64.3%)	286	(65.4%)	0.110
ICT and/or ICPs record the species and trends of isolated microorganisms on a type-of-sample and a ward-by-ward basis.	636	(93.8%)	530	(94.8%)	0.142	413	(94.5%)	414	(94.7%)	0.257
We have a direct and fast reporting system to the doctor-in-charge, such as e-mail and telephone, when microorganisms are isolated from a sample that is supposed to be aseptic (e.g., a blood sample).	653	(96.3%)	550	(98.4%)	0.068	422	(96.6%)	431	(98.6%)	0.088
**We perform surveillance for surgical site infections.**	510	(75.2%)	446	(79.8%)	**0.038**	334	(76.4%)	355	(81.2%)	0.119
**We perform surveillance for ventilator-associated pneumonia.**	238	(35.1%)	219	(39.2%)	0.254	162	(37.1%)	175	(40.0%)	0.422
**We perform surveillance for central line-associated bloodstream infections.**	508	(74.9%)	440	(78.7%)	0.190	330	(75.5%)	351	(80.3%)	0.088
**We perform surveillance for catheter-associated urinary tract infections.**	345	(50.9%)	310	(55.5%)	0.275	224	(51.3%)	258	(59.0%)	0.063
**We perform active surveillance cultures.**	334	(49.3%)	273	(48.8%)	0.905	228	(52.2%)	219	(50.1%)	0.831
We have an established manual for outbreaks.	637	(94.0%)	534	(95.5%)	0.370	417	(95.4%)	419	(95.9%)	0.947
**3. Preventive measures by the route of infections**										
We have a manual for the outbreak of tuberculosis.	675	(99.6%)	559	(100.0%)	0.290	435	(99.5%)	437	(100.0%)	0.368
We have a manual for the outbreak of measles.	623	(91.9%)	513	(91.8%)	0.175	398	(91.1%)	401	(91.8%)	0.222
We have a manual for the outbreak of chickenpox.	612	(90.3%)	502	(89.8%)	0.161	393	(89.9%)	395	(90.4%)	0.222
We provide N95 masks at the outpatient emergency department and other outpatient departments.	664	(97.9%)	551	(98.6%)	0.648	429	(98.2%)	432	(98.9%)	0.661
We put a surgical mask on patients with suspected airborne infections while transporting.	677	(99.9%)	558	(99.8%)	0.361	436	(99.8%)	436	(99.8%)	0.368
Wearing an N95 mask is mandatory while entering the ward of a patient with suspected tuberculosis.	676	(99.7%)	558	(99.8%)	0.680	436	(99.8%)	436	(99.8%)	1.000
We have a manual for the outbreak of influenza.	674	(99.4%)	555	(99.3%)	0.736	435	(99.5%)	435	(99.5%)	1.000
Wearing a surgical mask while entering the ward of a patient with a droplet infection is instructed by a manual.	671	(99.0%)	558	(99.8%)	0.152	432	(98.9%)	437	(100.0%)	0.081
We provide surgical masks in the wards of patients with droplet infections.	589	(86.9%)	486	(86.9%)	0.716	374	(85.6%)	380	(87.0%)	0.336
We have a manual for cases in which MRSA is isolated from a patient.	667	(98.4%)	551	(98.6%)	0.661	429	(98.2%)	433	(99.1%)	0.400
Wearing disposable gloves and a gown is mandatory while entering the ward of a patient with suspected contagious diseases.	618	(91.2%)	508	(90.9%)	0.966	399	(91.3%)	401	(91.8%)	0.793
We provide alcohol-based hand sanitizers in all wards except for some special wards, such as the psychiatric ward.	657	(96.9%)	546	(97.7%)	0.525	427	(97.7%)	428	(97.9%)	0.607
We provide alcohol-based hand sanitizers in all outpatient departments.	624	(92.0%)	529	(94.6%)	0.151	404	(92.4%)	415	(95.0%)	0.224
**4. Maintenance of medical equipment**										
We adopt closed urine drainage systems.	644	(95.0%)	544	(97.3%)	0.112	419	(95.9%)	426	(97.5%)	0.412
**We do not change catheters without blockages or infections regularly.**	512	(75.5%)	418	(74.8%)	0.619	322	(73.7%)	323	(73.9%)	0.904
We have a manual for the maintenance of ventilators.	583	(86.0%)	499	(89.3%)	0.221	376	(86.0%)	388	(88.8%)	0.424
We adopt closed tracheal suction systems.	568	(83.8%)	476	(85.2%)	0.799	382	(87.4%)	381	(87.2%)	0.931
We use sterile water for humidifiers.	658	(97.1%)	544	(97.3%)	0.120	428	(97.9%)	426	(97.5%)	0.311
**We perform regular oral cleansing for intubated patients in approximately 100% of relevant cases.**	524	(77.3%)	425	(76.0%)	0.225	340	(77.8%)	333	(76.2%)	0.226
We have a manual for the maintenance of central line catheters.	654	(96.5%)	542	(97.0%)	0.108	418	(95.7%)	425	(97.3%)	0.294
**We insert central line catheters under maximal barrier precautions in approximately 100% of relevant cases.**	254	(37.5%)	210	(37.6%)	0.086	163	(37.3%)	167	(38.2%)	0.150
**We prepare intravenous hyperalimentation admixtures on clean benches in approximately 100% of relevant cases.**	277	(40.9%)	225	(40.3%)	0.415	182	(41.6%)	175	(40.0%)	0.335
We use transparent dressings on the sites of catheter insertion to make them easy to inspect visually in approximately 100% of relevant cases.	563	(83.0%)	486	(86.9%)	0.224	357	(81.7%)	380	(87.0%)	0.112
**5. Standard precautions**										
**We instruct new employees in hand hygiene by practical training sessions for all professions.**	361	(53.2%)	290	(51.9%)	0.955	229	(52.4%)	222	(50.8%)	0.700
We evaluate the implementation of hand hygiene instructions of all wards at least once a year.	603	(88.9%)	523	(93.6%)	**0.018**	389	(89.0%)	411	(94.1%)	**0.028**
**We instruct new employees of all professions how to put on and remove PPE.**	532	(78.5%)	426	(76.2%)	0.638	347	(79.4%)	330	(75.5%)	0.255
**We instruct all employees in PPE by practical training sessions every year.**	135	(19.9%)	107	(19.1%)	0.281	85	(19.5%)	80	(18.3%)	0.126
**6. Wards**										
We provide hand sanitizers at the entrance of all wards.	656	(96.8%)	544	(97.3%)	0.407	426	(97.5%)	426	(97.5%)	0.593
All medical devices (e.g., thermometers, stethoscopes) of single isolation rooms are patient-dedicated.	653	(96.3%)	529	(94.6%)	0.152	423	(96.8%)	414	(94.7%)	0.174
We check expiry dates of sterilized medical devices daily.	638	(94.1%)	528	(94.5%)	0.949	415	(95.0%)	416	(95.2%)	0.987
We check expiry dates of unused medications.	664	(97.9%)	551	(98.6%)	0.516	429	(98.2%)	430	(98.4%)	0.741
We have established guides for the expiry dates of opened medications.	649	(95.7%)	542	(97.0%)	0.285	421	(96.3%)	422	(96.6%)	0.514
All wards have at least one infection control link nurse.	669	(98.7%)	547	(97.9%)	0.535	432	(98.9%)	429	(98.2%)	0.571
**7. ICU**										
**Medical professions do not change their shoes while entering ICU.**	548	(80.8%)	425	(76.0%)	0.123	363	(83.1%)	335	(76.7%)	**0.037**
**Medical professions are not recommended to wear gowns while entering ICU.**	548	(80.8%)	425	(76.0%)	0.116	361	(82.6%)	337	(77.1%)	0.128
**We have handwashing sinks at the entrance of ICU.**	397	(58.6%)	320	(57.2%)	0.085	259	(59.3%)	248	(56.8%)	0.107
**We provide hand sanitizers at the entrance of ICU.**	549	(81.0%)	426	(76.2%)	0.114	362	(82.8%)	338	(77.3%)	0.095
**We advise the patients’ families to use hand sanitizers or wash hands before and after entering ICU.**	545	(80.4%)	428	(76.6%)	**0.016**	362	(82.8%)	339	(77.6%)	0.066
**8. Operating room**										
We do not change stretchers while entering operating rooms.	518	(76.4%)	449	(80.3%)	**0.046**	334	(76.4%)	352	(80.5%)	0.211
**Medical professions do not change their shoes while entering operating rooms.**	395	(58.3%)	356	(63.7%)	0.102	263	(60.2%)	285	(65.2%)	0.299
We do not provide sticky mats at the entrance of operation rooms.	670	(98.8%)	552	(98.7%)	0.734	434	(99.3%)	432	(98.9%)	0.715
We have established standards of surgical hand preparation.	579	(85.4%)	492	(88.0%)	0.331	375	(85.8%)	381	(87.2%)	0.553
We do not recommend the use of a brush for surgical hand preparation.	641	(94.5%)	534	(95.5%)	0.424	419	(95.9%)	420	(96.1%)	0.867
**9. Prevention of postoperative infections**										
We use electric clippers or depilatory creams for patients who need to remove their hair before surgery in all departments.	651	(96.0%)	532	(95.2%)	0.572	420	(96.1%)	418	(95.7%)	0.271
We advise patients who can take a shower to take a shower on the night before or the morning of the day of surgery.	638	(94.1%)	526	(94.1%)	0.865	410	(93.8%)	410	(93.8%)	0.478
We recommend the administration of prophylactic antibiotics 30 min to 1 h before the incision.	640	(94.4%)	522	(93.4%)	0.582	421	(96.3%)	406	(92.9%)	0.710
**We have manuals to establish the duration of prophylactic antibiotics administration in all departments.**	304	(44.8%)	266	(47.6%)	0.532	188	(43.0%)	214	(49.0%)	0.230
**10. Management of food hygiene in hospitals**										
We adopt dry kitchen systems for hospital meals.	508	(74.9%)	453	(81.0%)	**0.005**	330	(75.5%)	356	(81.5%)	**0.040**
**11. Management of medical waste**										
We distinguish infectious waste from other waste and store it in a place inaccessible to non-authorized people.	667	(98.4%)	546	(97.7%)	0.667	428	(97.9%)	427	(97.7%)	0.607
**12. Cleaning, disinfection, and sterilization of instruments**										
We do not pre-clean or pre-disinfect medical devices in wards.	549	(81.0%)	463	(82.8%)	0.526	355	(81.2%)	368	(84.2%)	0.501
We clean and disinfect endoscopes in accordance with the manuals or check them regularly.	582	(85.8%)	472	(84.4%)	0.498	375	(85.8%)	372	(85.1%)	0.885

*ICD* Infection control doctor, *MD* Medical doctor, *PhD* Doctor of philosophy, *ICT* Infection control team, *JANIS* Japan Nosocomial Infections Surveillance, *ICC* Infection control committee, *AST* Antimicrobial stewardship team, *MRSA* Methicillin-resistant *Staphylococcus aureus*, *TDM* Therapeutic drug monitoring, *HBsAg* Hepatitis B surface antigen, *IGRA* Interferon-gamma release assay, *ICP* Infection control practitioner, *PPE* Personal protective equipment, *ICU* Intensive care unit

Values are presented as medians (interquartile range) for numeric variables and numbers (%) for categorical variables

Questions in bold indicate that the proportion of the most favorable answer was < 80%

*Student’s t-test or Satterthwaite test as appropriate for continuous variables; Cochran-Mantel-Haenszel test for categorical variables

*P* values in bold indicate *P* < .05

More than 90% of hospitals had weekly ICT meetings, although proportions of specific activities differed from hospital to hospital (section 2): 79.9% (1st survey) and 66.7% (2nd survey) of hospitals had an antimicrobial stewardship team. More than 90% of hospitals indicated that they monitored and intervened to assure appropriate use of antibiotics, but only 70% had established intervention criteria. The proportions of hospitals with intervention criteria for patients administered anti-methicillin-resistant *Staphylococcus aureus* (MRSA) antibiotics and carbapenems were approximately 60 and 50%, respectively. The proportions of hospitals that performed surveillance varied by the types of infections: catheter-associated urinary tract infections and ventilator-associated pneumonia were monitored less frequently compared to surgical site infections and central line-associated bloodstream infections.

With regard to the maintenance of medical equipment (section 4), less than 50% of hospitals indicated that they used maximal barrier precautions for central line catheter insertion and prepared intravenous hyperalimentation admixtures on clean benches.

For standard precautions (section 5), approximately 50% of hospitals held practical hand hygiene training sessions for new employees regardless of professions; the remaining hospitals trained new employees of selected professions only. Training regarding personal protective equipment for all new employees was held in about 80% of hospitals, although less than 20% of hospitals held these training sessions every year.

Regarding the ICU (section 7), the proportion of hospitals that answered “yes” to “We have handwashing sinks at the entrance of ICU” was lower than the other questions in this section. Roughly 60% of hospitals had handwashing sinks at the ICU entrance, whereas approximately 80% of hospitals answered “yes” for other questions.

Less than 70% of hospitals responded that their staff members do not change their shoes when entering the operating room, and less than 50% had manuals regarding the duration of prophylactic antibiotics available in all departments (section 8). The proportion was lower than 80% even when hospitals that had manuals in selected departments were included (Tables S1 and S2 in Additional file 2).

Table 2 presents the proportions of isolated microorganisms and antimicrobial-resistant microorganisms. Among antimicrobial-resistant microorganisms, only the proportion of those belonging to the family Enterobacteriaceae decreased in 2016 compared with 2015. The proportions of antimicrobial-resistant *Streptococcus pneumoniae* and *Escherichia coli* increased during this period.

**Table 2:** Isolation proportions of microorganisms and antimicrobial-resistant microorganisms

	Number of hospitals	1st survey (2015)				2nd survey (2016)				
Microorganism		Mean	±SD	Median	(IQR)	Mean	±SD	Median	(IQR)	P*
***Staphylococcus aureus***	378	14.4%	±7.7%	13.2%	(9.6–17.3%)	15.0%	±7.4%	14.2%	(9.7–19.0%)	**0.046**
Methicillin-resistant	378	37.8%	±14.3%	34.9%	(28.6–43.9%)	37.9%	±13.3%	35.8%	(28.6–44.7%)	0.342
Methicillin-resistant, in a blood sample	378	6.0%	±4.3%	5.1%	(3.2–8.1%)	6.8%	±4.9%	6.2%	(3.9–8.8%)	**0.002**
***Streptococcus pneumoniae***	296	2.1%	±2.3%	1.5%	(0.8–2.7%)	1.9%	±2.7%	1.2%	(0.7–2.1%)	**<.001**
Penicillin-resistant	296	22.6%	±21.9%	18.9%	(0.3–40.4%)	29.1%	±20.8%	33.3%	(5.5–45.5%)	**<.001**
***Escherichia coli***	298	12.8%	±7.3%	11.8%	(8.3–15.7%)	12.6%	±5.9%	11.6%	(9.0–15.4%)	0.181
Fluoroquinolone-resistant	298	27.2%	±11.0%	27.8%	(19.7–34.4%)	29.4%	±10.6%	30.0%	(22.0–36.4%)	**<.001**
***Pseudomonas aeruginosa***	299	4.7%	±3.3%	4.1%	(2.7–5.8%)	5.2%	±2.9%	4.8%	(3.4–6.4%)	**<.001**
Carbapenem-resistant	299	10.8%	±7.2%	9.8%	(6.0–14.8%)	10.8%	±7.1%	10.0%	(5.1–15.6%)	0.843
**Enterobacteriaceae**	279	22.5%	±10.9%	21.2%	(15.2–28.0%)	18.6%	±9.3%	17.2%	(13.2–22.9%)	**<.001**
Carbapenem-resistant	279	1.0%	±1.5%	0.5%	(0.1–1.4%)	0.9%	±1.7%	0.2%	(0.0–0.9%)	**0.001**

*SD* Standard deviation, *IQR* Interquartile range

*Wilcoxon signed-rank test

*P* values in bold indicate *P* < .05

Tables 3, 4, 5, 6, 7, and Table S3 and Figure S1 in Additional file 2 show the results of the LTA. Five statutes, from the most favorable (status 1) to the least favorable (status 5), were identified (Table 3). Latent status 4 showed the highest status membership probabilities for both time periods (Table 4). As for transition probabilities, members of statuses 1, 2, 4, and 5 were stable in their status membership (Table 5). On the other hand, members of status 3 showed the lowest probability of remaining in the same status (42.7%, Table 5), with 32.7% moving to status 5 and 14.8% moving to status 4 (Table 5). We assigned five domains according to the item-response probabilities for each question (Tables 3, 6 and Figure S1 in Additional file 2): “antimicrobial stewardship” (domain 1); “surveillance” (domain 2); “medical and hospital equipment” (domain 3); “ICT activities regarding vaccinations and education of employees” (domain 4); and “acknowledgment of updating relevant guidelines” (domain 5). Compared to status 1, status 2 showed lower probabilities of having criteria for anti-MRSA antibiotic use and broad-spectrum antibiotic use, whereas status 3 had lower probabilities of performing surveillance. Status 4 had only one domain (i.e., domain 3) with higher probabilities for questions in it. Status 5 had no domain that showed higher probabilities compared to other statuses. In the analysis using the number of ICT members (FTE per 100 beds) as a covariate, the odds ratio of status 3 versus status 5 was 1.32, whereas odds ratios were 0.55 and 0.61 for statuses 1 and 2 versus status 5, respectively (*p* = 0.027, Table 7).

**Table 3:** Item-response probabilities for each question by identified latent statuses

Domain	Question	Latent status				
		1	2	3	4	5
Antimicrobial stewardship	We have an antimicrobial stewardship team (a member can work for both an infection control team and an antimicrobial stewardship team).	**0.853**	**0.828**	**0.934**	0.742	0.743
	We have established criteria of interventions, such as their administration duration and selection, for patients administered antibiotics.	**0.819**	**0.729**	**0.821**	0.653	0.563
	We have criteria for the uses of anti-MRSA antibiotics.	**1**	0	**1**	0.595	0.487
	We have a preauthorization and/or restriction system for the use of anti-MRSA antibiotics.	**0.525**	**0.505**	**1**	0.176	0.169
	We have criteria for the uses of broad-spectrum antibiotics such as carbapenems.	**0.847**	0.187	**0.856**	0.385	0.304
	We have a preauthorization and/or restriction system for the use of broad-spectrum antibiotics.	**0.365**	0.348	**0.947**	0.072	0.065
	We performed therapeutic drug monitoring for basically all cases.	**0.800**	**0.723**	0.607	0.593	0.506
	We have manuals to establish the duration of prophylactic antibiotics administration in all departments.	**0.603**	**0.621**	**0.513**	0.426	0.324
Surveillance	We perform surveillance for ventilator-associated pneumonia.	**0.819**	**0.780**	0.164	0.114	0.037
	We perform surveillance for catheter-associated urinary tract infections.	**0.874**	**0.710**	0.261	0.450	0.375
	We perform active surveillance cultures.	**0.798**	**0.738**	0.315	0.402	0.298
Medical and hospital equipment	We perform regular oral cleansing for intubated patients in approximately 100% of relevant cases.	**0.816**	**0.808**	0.749	**0.801**	0.712
	We insert central line catheters under maximal barrier precautions in approximately 100% of relevant cases.	0.374	0.358	0.416	**1**	0.000
	We prepare intravenous hyperalimentation admixtures on clean benches in approximately 100% of relevant cases.	0.400	0.393	**0.496**	**0.521**	0.340
	We have handwashing sinks at the entrance of an intensive care unit.	**0.673**	**0.687**	0.476	0.502	0.533
Infection control team activities regarding vaccinations and education of employees	Needle puncture and sharp object injuries are reported to a relevant department, such as an infection control team.	**0.735**	0.580	**0.756**	**0.805**	0.644
	We instruct new employees in hand hygiene by practical training for all professions.	0.493	0.423	**0.593**	0.460	**0.571**
	We instruct new employees of all professions how to put on and remove personal protective equipment.	0.762	0.745	**0.818**	**0.774**	0.769
	We instruct all employees in personal protective equipment by practical training every year.	**0.198**	0.125	**0.242**	0.146	**0.202**
Acknowledgment of updating relevant guidelines	We do not change catheters without blockages or infections regularly.	**0.892**	**0.786**	0.657	0.666	0.657
	Medical professions do not change their shoes while entering operating rooms.	**0.764**	**0.713**	0.509	0.532	0.509

Values in bold indicate that the probability was above the mean of each question (i.e., each row)

**Table 4:** Status membership probabilities for the 1st and 2nd time periods

Time	Latent status				
	1	2	3	4	5
**1**	0.236	0.171	0.180	0.149	0.263
**2**	0.253	0.171	0.088	0.170	0.318

**Table 5:** Transition probabilities of each status from the 1st to 2nd time periods

		Status, time 2				
		1	2	3	4	5
**Status, time 1**	**1**	0.996	0	0	0.004	0
	**2**	0	1	0	0	0
	**3**	0.099	0	0.427	0.148	0.327
	**4**	0	0	0.040	0.961	0
	**5**	0	0	0.015	0	0.985

**Table 6:** Characteristics of each latent status

	Latent status				
Domain, number of questions in each domain	1	2	3	4	5
Antimicrobial stewardship, 8	✓	✓	✓	*✗*	*✗*
Surveillance, 3	✓	✓	*✗*	*✗*	*✗*
Medical and hospital equipment, 4			*✗*	✓	*✗*
Infection control team activities regarding vaccinations and education of employees, 4		*✗*	✓		
Acknowledgment of updating relevant guidelines, 2	✓	✓	*✗*	*✗*	*✗*

✓, the number of questions for which the probability (shown in Table 3) was above the mean of probabilities for five domains for each question (the mean value of each row in Table 3) was higher than half of the number of questions in each domain; *✗*, the number of questions for which the probability (shown in Table 3) was above the mean of probabilities for five domains for each question (the mean value of each row in Table 3) was lower than a half of the number of questions in each domain. For example, latent status 2 had 7 questions of which probabilities were higher than the mean probability of corresponding questions in the first domain “antimicrobial stewardship.” These 7 probabilities were written in bold. The number of these bold ones was 5, which was more than half of the number of questions in the domain “antimicrobial stewardship,” 8, then status 3 was assigned to “✓ “for the first domain

**Table 7:** Odds ratio estimates of covariates

Covariate	Latent status					*P*
	1	2	3	4	5	
Number of members in an infection control team*	0.55	0.61	1.32	0.74	Reference	0.027

* In full-time equivalent per 100 hospital beds

## Discussion

We conducted two surveys on AMR and infections in teaching hospitals in Japan, with an interval of approximately 1 year between the surveys. Most hospitals had activities of ICTs, however, actual activities differed among hospitals. The results of LTA suggested that there were five subgroups of hospitals, which were considered indicating similar achievement levels of AMS. The presence of local (i.e. hospital-level) guidelines for using anti-MRSA and broad-spectrum antibiotics, and the range of surveillance activities of each hospital were identified as two major determinants of the membership in each subgroup.

The proportion of hospitals with antimicrobial stewardship teams decreased during the study period. In fiscal year 2018 (after the 2nd survey), a fee for antimicrobial stewardship teams was introduced by the National Fee Schedule. To claim this fee, hospitals must fulfill requirements such as having at least one full-time staff member who is a physician with more than 3 years of experience in infectious disease treatment, a nurse with more than 5 years of experience working in a hospital, or a pharmacist or a laboratory technologist with more than 3 years of experience working in a hospital. Our results suggest that hospitals not fulfilling this requirement might have changed their answers to this question from “having an antimicrobial stewardship team” to “not having an antimicrobial stewardship team.”

The proportion of hospitals with preauthorization and/or restriction systems for the use of anti-MRSA antibiotics and broad-spectrum antibiotics decreased during the study period. Preauthorization and/or prospective audit and feedback interventions by AMS programs are strongly recommended [11]. Although more than 90% of hospitals in our study responded that they carried out monitoring and intervention activities, roughly 70% had established intervention criteria, and less than 40% had preauthorization and/or restriction systems for anti-MRSA antibiotics and broad-spectrum antibiotics. These proportions also decreased throughout the study period. The use of restricted antibiotics lists has been reported to reduce antimicrobial resistance rates and costs [12]. Thus, hospitals should consider introducing preauthorization and/or restriction systems for relevant antibiotics to enhance their AMS programs.

The proportions of hospitals with surveillance for ventilator-associated pneumonia and catheter-associated urinary tract infections increased slightly, but remained under 60%. Given that these infections are considered major healthcare-associated infections along with surgical site infections and central line-associated bloodstream infections, surveillance is recommended [13–15]. The proportion of hospitals performing active surveillance cultures was roughly 65%. However, active surveillance cultures for MRSA and vancomycin-resistant enterococci for all inpatients except for high-risk patients are not recommended [16]. The WHO Guidelines Development Group strongly recommends surveillance cultures for asymptomatic carbapenem-resistant Enterobacteriaceae and surveillance for carbapenem-resistant *Acinetobacter baumannii* and *Pseudomonas aeruginosa* despite a very low quality of evidence [17]. Further studies will be needed to determine the targets for active surveillance cultures and their efficacy.

For all questions regarding the ICU, the proportions of hospitals with the most favorable answers were less than 80%. This might be due to the fact that hospitals without an ICU were also included in this study. However, the proportion of hospitals that answered “yes” to the question about handwashing sinks at the ICU entrance was considerably lower (less than 60%) compared to those of hospitals that answered “yes” to the other questions. A Japanese guideline (2002) that recommended hospitals to place handwashing sinks at the ICU entrance [18] was revised to allow for the location to be based on staff accessibility [19]. However, since recent studies have suggested that sinks in the ICU might be a source of infections [20–22], further investigations will be needed on appropriate locations and specifications of sinks in the ICU.

The LTA identified five statuses. There was a slight increase in the most favorable status (status 1) over the course of the study period (23.6 to 25.3%). However, the least favorable status (status 5) also showed an increase (26.3 to 31.8%), which was mainly due to a decrease in status 3 (18.0 to 8.8%). Previous studies have reported that human resources (FTEs of infection control practitioners) and FTE-to-bed ratios were related to improvements in AMS [3–5], defined as an increase in the number of implemented AMS programs [3] or effectiveness of AMS programs [4]. However, improvements in AMS may not correlate with the number of implemented programs, considering that the weight of each program is unlikely to be equal. In fact, the results of the LTA do not support a relationship between increasing the number of FTEs per bed and being in more favorable latent statuses. The odds ratio of status 3 versus status 5 was 1.32, indicating that more infection control practitioners might be required to improve domain 1, “antimicrobial stewardship,” whereas improvement in domains 2, 3, and 5 could not be fully explained by an increase in human resources alone. However, since previous studies, as well as our study, did not account for patient-level variations, further studies will be needed to identify factors associated with AMS other than human resources.

This study had some limitations. First, response rates were 55.0% for all hospitals with valid responses and 43.0% for those that responded to both surveys. There may have been selection bias in these hospitals. Second, hospitals participating in our study may have different profiles of cases and individual risks. To address these issues, we plan to link administrative data and the data of this study for further analyses.

## Conclusion

The present nationwide surveys revealed the need for more comprehensive AMS programs; specifically, hospitals should consider introducing preauthorization and/or restriction systems for anti-MRSA antibiotics and broad-spectrum antibiotics. Our results also suggest that surveillance activities for ventilator-associated pneumonia and catheter-associated urinary tract infections need to be increased.

## Supplementary Information

**Additional file 1.** English translation of the questionnaire for the study.

**Additional file 2: Supplementary tables and a figure.**

## Data Availability

The datasets used and/or analyzed during the current study are de-identified and available from the corresponding author on reasonable request.

## Abbreviations

AMR: Antimicrobial resistance
AMS: Antimicrobial stewardship
FTE: Full-time equivalent
ICT: Infection control team
ICU: Intensive care unit
LTA: Latent transition analysis
MRSA: Methicillin-resistant *Staphylococcus aureus*
