# Supplementary material for: Nationwide multicenter questionnaire surveys on countermeasures against antimicrobial resistance and infections in hospitals
Source: BMC Infect Dis. 2021 Feb 27;21:234. doi: 10.1186/s12879-021-05921-2 (PMC7912490; doi:10.1186/s12879-021-05921-2)
Supplement: Supplementary file 1 — Additional file 1. English translation of the questionnaire for the study. [file 12879_2021_5921_MOESM1_ESM.docx]

Additional file 1. Questionnaire for the study (English translation)

Survey on countermeasures against nosocomial infections

# General information

## Number of hospital beds (first survey only)

General beds: _____

Long-term care beds: _____

Beds for psychiatric diseases: _____

Beds for tuberculosis: _____

Beds for infectious diseases: _____

## Our hospital is *(first survey only)*

□ a designated medical institution for specific infectious diseases

□ a designated medical institution for type I infectious diseases

□ a designated medical institution for type II infectious diseases

□ a designated medical institution for tuberculosis

□ not a designated medical institution for infectious diseases

## Our hospital is eligible to claim *(first survey only)*

□ a fee for countermeasures for nosocomial infections, type 1

□ a fee for countermeasures for nosocomial infections, type 2

□ a fee for regional cooperation on countermeasures for nosocomial infections

## We performed bacterial culture, identification, and susceptibility tests basically in our hospital.

□ Yes □ No

## Number of staff

Physician (full-time): _____

Infectious disease specialist: _____

Infection control trainer: _____

Certified microbiologist: _____

Certified infection control doctor: _____

Nurse (full-time): _____

Certified nurse in infection control: _____

Professional nurse for infection prevention and control: _____

Certified nurse specialist in infection control nursing: _____

Master of infection control-related course: _____

Laboratory technologist (full-time): _____

Certified laboratory technologist in infection control: _____

Pharmacist (full-time): _____

Infection control pharmacy specialist: _____

Certified pharmacist in infection control: _____

Infectious disease chemotherapy pharmacist: _____

Dietitian: _____

Administrative staff: _____

Certified infection control doctor (MD or PhD): _____

## Does your hospital have an infection control team (ICT)?

□ Yes □ No

If yes, please provide details below:

Number of ICT member, crude

Total: _____

Physician: _____

devoting ≥50% of working time to the ICT: _____

devoting ≥80% of working time to the ICT: _____

≥3-years’ experience of infection control measures: _____

Nurse: _____

devoting ≥50% of working time to the ICT: _____

devoting ≥80% of working time to the ICT: _____

≥5-years’ experience of infection control measures: _____

Pharmacist: _____

devoting ≥50% of working time to the ICT: _____

devoting ≥80% of working time to the ICT: _____

≥3-years’ experience as a hospital pharmacist: _____

Laboratory technologist: _____

devoting ≥50% of working time to the ICT: _____

devoting ≥80% of working time to the ICT: _____

≥3-years’ experience as a hospital technologist: _____

Dietitian: _____

devoting ≥50% of working time to the ICT: _____

devoting ≥80% of working time to the ICT: _____

Administrative staff: _____

devoting ≥50% of working time to the ICT: _____

devoting ≥80% of working time to the ICT: _____

## We participate in JANIS (Japan Nosocomial Infections Surveillance) programs.

□ Yes □ No

If yes, please check the appropriate boxes:

□ Clinical laboratory division

□ Antimicrobial-resistant bacterial infection division

□ Surgical site infection division

□ Intensive care unit division

□ Neonatal intensive care unit division

# 1. Organizational structure for nosocomial infection control

## The head of our hospital attends the infection control committee almost every time.

□ Yes □ No

## We have a comprehensive hospital infection control manual that can be used all around our hospital.

□ Yes □ No

## We hold a workshop regarding countermeasures against hospital infection more than once a year.

□ Yes □ No

## We have tools, such as the intranet and bulletin boards, to inform our staff of hospital infection-related matters.

□ Yes □ No

# 2. Activities of ICT

## We hold a regular ICT meeting.

□ Yes □ No

## We provide consultation as an activity of the ICT.

□ Yes □ No

## We have an antimicrobial stewardship team (AST; a member can work for both ICT and AST).

□ Yes □ No

## We monitor the uses of antibiotics to assure their propriety.

□ Yes □ No

## We intervene to assure appropriate uses of antibiotics.

□ Yes □ No

## We have established criteria of interventions, such as their administration duration and selection, for patients administered antibiotics.

□ Yes □ No

## We have criteria for the uses of anti- methicillin-resistant Staphylococcus aureus (MRSA) antibiotics.

□ Yes □ No

## We record the used amount of anti-MRSA antibiotics.

□ Yes □ No

## We have a reporting system (first survey: “*registration* system”) for the use of anti-MRSA antibiotics.

□ Yes □ No

## We have a preauthorization and/or restriction system for the use of anti-MRSA antibiotics.

□ Yes □ No

## We have criteria for the uses of broad-spectrum antibiotics such as carbapenems.

□ Yes □ No

## We have a reporting system (first survey: “*registration* system”) for the use of broad-spectrum antibiotics.

□ Yes □ No

## We have a preauthorization and/or restriction system for the use of broad-spectrum antibiotics.

□ Yes □ No

## We record the used amount of broad-spectrum antibiotics.

□ Yes □ No

## We have a reference system, such as the intranet of a booklet, for the antibiogram.

□ Yes □ No

## We performed therapeutic drug monitoring

□ basically all cases.

□ selected cases.

□ no cases.

## We record the vaccination proportion of employees who are HBsAg-negative.

□ Yes □ No

## We perform interferon-gamma release assays for employees who are in contact with tuberculosis patients.

## We record employees’ immunization statuses for measles, rubella, chickenpox, and mumps (second survey: “for *all of* measles, rubella, chickenpox, and mumps”).

□ Yes □ No

## We have a manual and a reporting system of needle punctures and sharp object injuries.

□ Yes □ No

## Needle puncture and sharp object injuries are reported to a relevant department, such as ICT

□ in approximately 100% of relevant cases.

□ in approximately 80% of relevant cases.

□ in approximately 50% of relevant cases.

□ in approximately 20% of relevant cases.

□ in approximately 0% of relevant cases.

## ICT and/or infection control practitioners check the number of isolated antimicrobial-resistant organisms and other microorganisms that are relevant to infection control

□ on a daily basis.

□ regularly.

□ We do not have such activities.

## ICT and/or ICPs record the species and trends of isolated microorganisms on a type-of-sample and a ward-by-ward basis.

□ Yes □ No

## We have a direct and fast reporting system to the doctor-in-charge, such as e-mail and telephone, when microorganisms are isolated from a sample that is supposed to be aseptic (e.g., a blood sample).

□ Yes □ No

## We perform surveillance for surgical site infections.

□ Yes □ No

## We perform surveillance for ventilator-associated pneumonia.

□ Yes □ No

## We perform surveillance for central line-associated bloodstream infections.

□ Yes □ No

## We perform surveillance for catheter-associated urinary tract infections.

□ Yes □ No

## We perform active surveillance cultures.

□ Yes □ No

## We have an established manual for outbreaks.

□ Yes □ No

# 3. Preventive measures by the route of infections

## We have a manual for the outbreak of tuberculosis.

□ Yes □ No

## We have a manual for the outbreak of measles.

□ Yes □ No

## We have a manual for the outbreak of chickenpox.

□ Yes □ No

## We provide N95 masks at the outpatient emergency department and other outpatient departments.

□ Yes □ No

## We put a surgical mask on patients with suspected airborne infections while transporting.

□ Yes □ No

## Wearing an N95 mask is mandatory while entering the ward of a patient with suspected tuberculosis.

□ Yes □ No

## We have a manual for the outbreak of influenza.

□ Yes □ No

## Wearing a surgical mask while entering the ward of a patient with a droplet infection is instructed by a manual.

□ Yes □ No

## We provide surgical masks in the wards of patients with droplet infections.

□ Yes □ No

## We have a manual for cases in which MRSA is isolated from a patient.

□ Yes □ No

## Wearing disposable gloves and a gown is mandatory while entering the ward of a patient with suspected contagious diseases.

□ Yes □ No

## We provide alcohol-based hand sanitizers in all wards except for some special wards, such as the psychiatric ward.

□ Yes □ No

## We provide alcohol-based hand sanitizers in all outpatient departments.

□ Yes □ No

# 4. Maintenance of medical equipment

## We adopt closed urine drainage systems.

□ Yes □ No

## We do not change catheters without blockages or infections regularly.

□ Yes □ No

## We have a manual for the maintenance of ventilators.

□ Yes □ No

## We adopt closed tracheal suction systems.

□ Yes □ No

## We use sterile water for humidifiers.

□ Yes □ No

## We perform regular oral cleansing for intubated patients in approximately 100% of relevant cases.

□ in approximately 100% of relevant cases.

□ in approximately 80% of relevant cases.

□ in approximately 50% of relevant cases.

□ in approximately 20% of relevant cases.

□ in approximately 0% of relevant cases.

## We have a manual for the maintenance of central line catheters.

□ Yes □ No

## We insert central line catheters under maximal barrier precautions

□ in approximately 100% of relevant cases.

□ in approximately 80% of relevant cases.

□ in approximately 50% of relevant cases.

□ in approximately 20% of relevant cases.

□ in approximately 0% of relevant cases.

## We prepare intravenous hyperalimentation admixtures on clean benches

□ in approximately 100% of relevant cases.

□ in approximately 80% of relevant cases.

□ in approximately 50% of relevant cases.

□ in approximately 20% of relevant cases.

□ in approximately 0% of relevant cases.

## We use transparent dressings on the sites of catheter insertion to make them easy to inspect visually

□ in approximately 100% of relevant cases.

□ in approximately 80% of relevant cases.

□ in approximately 50% of relevant cases.

□ in approximately 20% of relevant cases.

□ in approximately 0% of relevant cases.

# 5. Standard precautions

## We instruct new employees in hand hygiene by practical training sessions

□ for all professions.

□ for selected professions.

□ We do not have such training.

## We evaluate the implementation of hand hygiene instructions of all wards at least once a year.

□ Yes □ No

## We instruct new employees of all professions how to put on and remove personal protective equipment (PPE).

□ Yes □ No

## We instruct all employees in PPE by practical training sessions every year.

□ Yes □ No

# 6. Wards

## We provide hand sanitizers at the entrance of all wards.

□ Yes □ No

## All medical devices (e.g., thermometers, stethoscopes) of single isolation rooms are patient-dedicated.

□ Yes □ No

## We check expiry dates of sterilized medical devices daily.

□ Yes □ No

## We check expiry dates of unused medications.

□ Yes □ No

## We have established guides for the expiry dates of opened medications.

□ Yes □ No

## All wards have at least one infection control link nurse.

□ Yes □ No

# 7. Intensive care unit (ICU)

## Medical professions do not change their shoes while entering ICU.

□ Yes □ No

## Medical professions are not recommended to wear gowns while entering ICU.

□ Yes □ No

## We have handwashing sinks at the entrance of ICU.

□ Yes □ No

## We provide hand sanitizers at the entrance of ICU.

□ Yes □ No

## We advise the patients’ families to use hand sanitizers or wash hands before and after entering ICU.

□ Yes □ No

# 8. Operating room

## We do not change stretchers while entering operating rooms.

□ Yes □ No

## Medical professions do not change their shoes while entering operating rooms.

□ Yes □ No

## We do not provide sticky mats at the entrance of operation rooms.

□ Yes □ No

## We have established standards of surgical hand preparation.

□ Yes □ No

## We do not recommend the use of a brush for surgical hand preparation.

□ Yes □ No

# 9. Prevention of postoperative infections

## We use electric clippers or depilatory creams for patients who need to remove their hair before surgery in all departments.

□ Yes □ No

## We advise patients who can take a shower to take a shower on the night before or the morning of the day of surgery.

□ Yes □ No

## We recommend the administration of prophylactic antibiotics 30 minutes to 1 hour before the incision.

□ Yes □ No

## We have manuals to establish the duration of prophylactic antibiotics administration

□ in all departments.

□ in selected departments.

□ We do not have such manuals.

# 10. Management of food hygiene in hospitals

## We adopt dry kitchen systems for hospital meals.

□ Yes □ No

# 11. Management of medical waste

## We distinguish infectious waste from other waste and store it in a place inaccessible to non-authorized people.

□ Yes □ No

# 12. Cleaning, disinfection, and sterilization of instruments

## We do not pre-clean or pre-disinfect medical devices in wards.

□ Yes □ No

## We clean and disinfect endoscopes in accordance with the manuals or check them regularly.

□ Yes □ No

# 13. Number of cases for antimicrobial-resistant microorganisms (First survey: cases in the fiscal year 2015; second survey: cases in fiscal year 2016)

## Number of cases (inpatients): _____

## Number of cases (outpatients): _____

## Number of cases with isolated mircoorganisms:

*Staphylococcus aureus*: _____

Methicillin-resistant: _____

Methicillin-resistant, in a blood sample: _____

*Streptococcus pneumoniae*: _____

Penicillin-resistant: _____

*Escherichia coli*: _____

Fluoroquinolone-resistant: _____

*Pseudomonas aeruginosa*: _____

Carbapenem-resistant: _____

Enterobacteriaceae: _____

Carbapenem-resistant: _____
