# Supplementary material for: Nationwide multicenter questionnaire surveys on countermeasures against antimicrobial resistance and infections in hospitals
Source: BMC Infect Dis. 2021 Feb 27;21:234. doi: 10.1186/s12879-021-05921-2 (PMC7912490; doi:10.1186/s12879-021-05921-2)
Supplement: Supplementary file 2 — Additional file 2: Supplementary tables and a figure. [file 12879_2021_5921_MOESM2_ESM.docx]

# Supporting information

Supplement to: Shin J, Mizuno S, Okuno T, Itoshima H, Sasaki N, Kunisawa S, Kaku M, Yoshida M, Yoshiaki G, Morii D, Shibayama K, Ohmagari N, Imanaka Y. Nationwide multicenter questionnaire surveys on countermeasures against antimicrobial resistance and infections in hospitals.

Contents

Table S1. Results of the 1st and 2nd questionnaire surveys (all hospitals with valid responses)

Table S2. Results of the 1st and 2nd questionnaire surveys (hospitals that responded to both surveys)

Table S3. Fit statistics of the latent status analysis

Figure S1. Item-response probabilities for each question by identified statuses.

# Table S1. Results of the 1st and 2nd questionnaire surveys (all hospitals with valid responses)

|  | 1st survey (n=437) | | | | | |  | 2nd survey (n=437) | | | | | |  |  |
| --- | --- | --- | --- | --- | --- | --- | --- | --- | --- | --- | --- | --- | --- | --- | --- |
| Question | Number of hospitals | Response rate | Mean | ±SD | Median | (IQR) |  | Number of hospitals | Response rate | Mean | ±SD | Median | (IQR) |  | *P** |
| Number of staff |  |  |  |  |  |  |  |  |  |  |  |  |  |  |  |
| Physician (full-time) | 414 | 94.7% | 124.4 | ±143.4 | 80 | (50–140) |  | 415 | 95.0% | 126.8 | ±127.3 | 81 | (50–137) |  | 0.805 |
| Infectious disease specialist | 327 | 74.8% | 0.8 | ±1.5 | 0 | (0–1) |  | 326 | 74.6% | 1.0 | ±2.3 | 0 | (0–1) |  | 0.300 |
| Infection control trainer | 318 | 72.8% | 0.5 | ±0.8 | 0 | (0–1) |  | 310 | 70.9% | 0.5 | ±1.0 | 0 | (0–1) |  | 0.258 |
| Certified microbiologist | 289 | 66.1% | 0.0 | ±0.3 | 0 | (0–0) |  | 285 | 65.2% | 0.1 | ±0.3 | 0 | (0–0) |  | 0.717 |
| Certified infection control doctor |  |  |  |  |  |  |  | 284 | 65.0% | 0.2 | ±1.6 | 0 | (0–0) |  |  |
| Nurse (full-time) | 405 | 92.7% | 427.3 | ±240.8 | 368 | (246–543) |  | 406 | 92.9% | 435.6 | ±250.3 | 371 | (251–561) |  | 0.629 |
| Certified nurse in infection control | 421 | 96.3% | 1.6 | ±0.9 | 2 | (1–2) |  | 420 | 96.1% | 1.7 | ±0.9 | 2 | (1–2) |  | 0.098 |
| Professional nurse for infection prevention and control | 316 | 72.3% | 0.2 | ±0.6 | 0 | (0–0) |  | 320 | 73.2% | 0.2 | ±0.8 | 0 | (0–0) |  | 0.496 |
| Certified nurse specialist in infection control nursing |  |  |  |  |  |  |  | 317 | 72.5% | 0.1 | ±0.3 | 0 | (0–0) |  |  |
| Master of infection control-related course | 311 | 71.2% | 0.1 | ±0.4 | 0 | (0–0) |  | 312 | 71.4% | 0.1 | ±0.3 | 0 | (0–0) |  | 0.190 |
| Laboratory technologist (full-time) | 420 | 96.1% | 30.3 | ±22.4 | 24 | (17–36.5) |  | 416 | 95.2% | 30.6 | ±21.2 | 24.5 | (17–37) |  | 0.819 |
| Certified laboratory technologist in infection control | 345 | 78.9% | 0.6 | ±0.9 | 0 | (0–1) |  | 346 | 79.2% | 0.6 | ±1.0 | ｓ0 | (0–1) |  | 0.272 |
| Pharmacist (full-time) | 418 | 95.7% | 24.3 | ±16.9 | 19 | (14–28) |  | 417 | 95.4% | 25.4 | ±17.7 | 20 | (14–30) |  | 0.360 |
| Infection control pharmacy specialist | 329 | 75.3% | 0.3 | ±0.6 | 0 | (0–0) |  | 319 | 73.0% | 0.3 | ±0.5 | 0 | (0–0) |  | 0.660 |
| Certified pharmacist in infection control | 355 | 81.2% | 0.7 | ±0.8 | 1 | (0–1) |  | 356 | 81.5% | 0.8 | ±0.9 | 1 | (0–1) |  | 0.077 |
| Infectious disease chemotherapy pharmacist | 329 | 75.3% | 0.6 | ±1.0 | 0 | (0–1) |  | 337 | 77.1% | 0.7 | ±0.9 | 0 | (0–1) |  | 0.471 |
| Dietitian | 415 | 95.0% | 6.3 | ±3.7 | 5 | (4–8) |  | 404 | 92.4% | 6.6 | ±4.0 | 6 | (4–8) |  | 0.370 |
| Administrative staff | 408 | 93.4% | 69.7 | ±56.7 | 56 | (33–87) |  | 405 | 92.7% | 71.2 | ±60.6 | 56 | (33–89) |  | 0.718 |
| Certified ICD (MD or PhD) | 380 | 87.0% | 3.1 | ±2.4 | 3 | (2–4) |  | 377 | 86.3% | 3.3 | ±2.5 | 3 | (2–4) |  | 0.322 |
| Number of ICT member, crude | 422 | 96.6% | 12.8 | ±7.7 | 11 | (8–17) |  | 415 | 95.0% | 13.2 | ±8.4 | 11 | (7–17) |  | 0.530 |
| Physician | 430 | 98.4% | 3.6 | ±3.3 | 3 | (2–4) |  | 433 | 99.1% | 3.7 | ±3.7 | 3 | (2–4) |  | 0.576 |
| devoting ≥50% of working time to the ICT | 430 | 98.4% | 1.3 | ±1.3 | 1 | (0–2) |  | 433 | 99.1% | 1.3 | ±1.3 | 1 | (0–2) |  | 0.984 |
| devoting ≥80% of working time to the ICT | 430 | 98.4% | 0.1 | ±0.4 | 0 | (0–0) |  | 433 | 99.1% | 0.1 | ±0.6 | 0 | (0–0) |  | 0.648 |
| ≥3-years’ experience of infection control measures | 430 | 98.4% | 2.4 | ±1.9 | 2 | (1–3) |  | 433 | 99.1% | 2.4 | ±2.2 | 2 | (1–3) |  | 0.657 |
| Nurse | 428 | 97.9% | 3.4 | ±3.3 | 2 | (2–4) |  | 435 | 99.5% | 3.5 | ±4.3 | 2 | (2–4) |  | 0.757 |
| devoting ≥50% of working time to the ICT | 428 | 97.9% | 0.5 | ±1.0 | 0 | (0–1) |  | 435 | 99.5% | 0.5 | ±1.0 | 0 | (0–1) |  | 0.627 |
| devoting ≥80% of working time to the ICT | 428 | 97.9% | 1.0 | ±0.6 | 1 | (1–1) |  | 435 | 99.5% | 1.1 | ±0.6 | 1 | (1–1) |  | 0.265 |
| ≥5-years’ experience of infection control measures | 428 | 97.9% | 1.8 | ±1.4 | 2 | (1–2) |  | 435 | 99.5% | 1.8 | ±1.4 | 2 | (1–2) |  | 0.472 |
| Pharmacist | 428 | 97.9% | 1.9 | ±0.9 | 2 | (1–2) |  | 432 | 98.9% | 2.0 | ±1.0 | 2 | (1–2) |  | 0.242 |
| devoting ≥50% of working time to the ICT | 428 | 97.9% | 1.0 | ±0.9 | 1 | (0–1) |  | 432 | 98.9% | 1.0 | ±0.8 | 1 | (0–1) |  | 0.818 |
| devoting ≥80% of working time to the ICT | 428 | 97.9% | 0.1 | ±0.3 | 0 | (0–0) |  | 432 | 98.9% | 0.0 | ±0.3 | 0 | (0–0) |  | 0.409 |
| ≥3-years’ experience as a hospital pharmacist | 428 | 97.9% | 1.7 | ±1.2 | 2 | (1–2) |  | 437 | 100.0% | 1.6 | ±1.0 | 2 | (1–2) |  | 0.545 |
| Laboratory technologist | 429 | 98.2% | 1.8 | ±0.9 | 2 | (1–2) |  | 431 | 98.6% | 1.8 | ±1.0 | 2 | (1–2) |  | 0.709 |
| devoting ≥50% of working time to the ICT | 429 | 98.2% | 1.0 | ±0.9 | 1 | (0–1) |  | 431 | 98.6% | 1.0 | ±0.8 | 1 | (0–1) |  | 0.878 |
| devoting ≥80% of working time to the ICT | 429 | 98.2% | 0.1 | ±0.3 | 0 | (0–0) |  | 431 | 98.6% | 0.1 | ±0.5 | 0 | (0–0) |  | 0.941 |
| ≥3-years’ experience as a hospital technologist | 429 | 98.2% | 1.6 | ±1.4 | 1 | (1–2) |  | 437 | 100.0% | 1.6 | ±1.0 | 1 | (1–2) |  | 0.566 |
| Dietitian | 368 | 84.2% | 0.2 | ±0.5 | 0 | (0–0) |  | 347 | 79.4% | 0.2 | ±0.5 | 0 | (0–0) |  | 0.948 |
| devoting ≥50% of working time to the ICT | 368 | 84.2% | 0.1 | ±0.3 | 0 | (0–0) |  | 347 | 79.4% | 0.0 | ±0.2 | 0 | (0–0) |  | 0.213 |
| devoting ≥80% of working time to the ICT | 368 | 84.2% | 0.0 | ±0.1 | 0 | (0–0) |  | 347 | 79.4% | 0.0 | ±0.0 | 0 | (0–0) |  | 0.083 |
| Administrative staff | 402 | 92.0% | 1.2 | ±1.1 | 1 | (0–2) |  | 394 | 90.2% | 1.2 | ±1.0 | 1 | (1–2) |  | 0.926 |
| devoting ≥50% of working time to the ICT | 402 | 92.0% | 0.3 | ±0.6 | 0 | (0–0) |  | 394 | 90.2% | 0.3 | ±0.6 | 0 | (0–0) |  | 0.966 |
| devoting ≥80% of working time to the ICT | 402 | 92.0% | 0.2 | ±0.4 | 0 | (0–0) |  | 394 | 90.2% | 0.1 | ±0.4 | 0 | (0–0) |  | 0.441 |
| Full time equivalent, all | 422 | 62.2% | 3.2 | ±2.1 | 2.8 | (1.6–4.3) |  | 415 | 74.2% | 3.2 | ±2.1 | 2.8 | (1.8–4.1) |  | 0.920 |
| Physician | 430 | 63.4% | 0.7 | ±0.7 | 0.5 | (0–1) |  | 433 | 77.5% | 0.7 | ±0.8 | 0.5 | (0.5–1) |  | 0.830 |
| Nurse | 428 | 63.1% | 1.1 | ±0.6 | 0.8 | (0.8–1.3) |  | 435 | 77.8% | 1.1 | ±0.6 | 0.8 | (0.8–1.3) |  | 0.693 |
| Pharmacist | 428 | 63.1% | 0.5 | ±0.5 | 0.5 | (0–0.8) |  | 432 | 77.3% | 0.5 | ±0.5 | 0.5 | (0–0.65) |  | 0.531 |
| Laboratory technologist | 429 | 63.3% | 0.5 | ±0.5 | 0.5 | (0–1) |  | 431 | 77.1% | 0.5 | ±0.5 | 0.5 | (0–0.8) |  | 0.931 |
| Dietitian | 368 | 54.3% | 0.0 | ±0.2 | 0 | (0–0) |  | 347 | 62.1% | 0.0 | ±0.1 | 0 | (0–0) |  | 0.067 |
| Administrative staff | 402 | 59.3% | 0.3 | ±0.4 | 0 | (0–0.5) |  | 394 | 70.5% | 0.2 | ±0.4 | 0 | (0–0.5) |  | 0.524 |

|  | 1st survey (n=437) | | | | | |  | 2nd survey (n=437) | | | | | |  |  |
| --- | --- | --- | --- | --- | --- | --- | --- | --- | --- | --- | --- | --- | --- | --- | --- |
| Question | Yes | | No | | No response | |  | Yes | | No | | No response | |  | *P** |
| We performed bacterial culture, identification, and susceptibility tests basically in our hospital. | 355 | (81.2%) | 71 | (16.2%) | 11 | (2.5%) |  | 367 | (84.0%) | 64 | (14.6%) | 6 | (1.4%) |  | 0.362 |
| We have an active ICT. | 436 | (99.8%) | 1 | (0.2%) | 0 | (0.0%) |  | 435 | (99.5%) | 1 | (0.2%) | 1 | (0.2%) |  | 0.607 |
| We participate in JANIS programs. | 426 | (97.5%) | 9 | (2.1%) | 2 | (0.5%) |  | 432 | (98.9%) | 3 | (0.7%) | 2 | (0.5%) |  | 0.219 |
| Clinical laboratory division | 421 | (96.3%) |  |  |  |  |  | 422 | (96.6%) |  |  |  |  |  | 0.855 |
| Antimicrobial-resistant bacterial infection division | 228 | (52.2%) |  |  |  |  |  | 235 | (53.8%) |  |  |  |  |  | 0.635 |
| Surgical site infection division | 249 | (57.0%) |  |  |  |  |  | 259 | (59.3%) |  |  |  |  |  | 0.493 |
| Intensive care unit division | 80 | (18.3%) |  |  |  |  |  | 74 | (16.9%) |  |  |  |  |  | 0.595 |
| Neonatal intensive care unit division | 56 | (12.8%) |  |  |  |  |  | 51 | (11.7%) |  |  |  |  |  | 0.606 |
| **1. Organizational structure for nosocomial infection control** |  |  |  |  |  |  |  |  |  |  |  |  |  |  |  |
| The head of our hospital attends ICC almost every time. | 379 | (86.7%) | 50 | (11.4%) | 8 | (1.8%) |  | 369 | (84.4%) | 67 | (15.3%) | 1 | (0.2%) |  | **0.018** |
| We have a comprehensive hospital infection control manual that can be used all around our hospital. | 437 | (100.0%) | 0 | (0.0%) | 0 | (0.0%) |  | 437 | (100.0%) | 0 | (0.0%) | 0 | (0.0%) |  | – |
| We hold a workshop regarding countermeasures against hospital infection more than once a year. | 437 | (100.0%) | 0 | (0.0%) | 0 | (0.0%) |  | 437 | (100.0%) | 0 | (0.0%) | 0 | (0.0%) |  | – |
| We have tools, such as the intranet and bulletin boards, to inform our staff of hospital infection-related matters. | 434 | (99.3%) | 3 | (0.7%) | 0 | (0.0%) |  | 436 | (99.8%) | 1 | (0.2%) | 0 | (0.0%) |  | 0.317 |
| **2. Activities of ICT** |  |  |  |  |  |  |  |  |  |  |  |  |  |  |  |
| We hold a regular ICT meeting. | 410 | (93.8%) | 27 | (6.2%) | 0 | (0.0%) |  | 416 | (95.2%) | 20 | (4.6%) | 1 | (0.2%) |  | 0.353 |
| We provide consultation as an activity of the ICT. | 412 | (94.3%) | 25 | (5.7%) | 0 | (0.0%) |  | 407 | (93.1%) | 28 | (6.4%) | 2 | (0.5%) |  | 0.333 |
| **We have an AST (a member can work for both ICT and AST).** | 355 | (81.2%) | 78 | (17.8%) | 4 | (0.9%) |  | 305 | (69.8%) | 126 | (28.8%) | 6 | (1.4%) |  | **<.001** |
| We monitor the uses of antibiotics to assure their propriety. | 420 | (96.1%) | 13 | (3.0%) | 4 | (0.9%) |  | 431 | (98.6%) | 5 | (1.1%) | 1 | (0.2%) |  | 0.064 |
| We intervene to assure appropriate uses of antibiotics. | 410 | (93.8%) | 19 | (4.3%) | 8 | (1.8%) |  | 415 | (95.0%) | 19 | (4.3%) | 3 | (0.7%) |  | 0.317 |
| **We have established criteria of interventions, such as their administration duration and selection, for patients administered antibiotics.** | 304 | (69.6%) | 121 | (27.7%) | 12 | (2.7%) |  | 310 | (70.9%) | 112 | (25.6%) | 15 | (3.4%) |  | 0.691 |
| **We have criteria for the uses of anti-MRSA antibiotics.** | 267 | (61.1%) | 164 | (37.5%) | 6 | (1.4%) |  | 278 | (63.6%) | 151 | (34.6%) | 8 | (1.8%) |  | 0.594 |
| We record the used amount of anti-MRSA antibiotics. | 432 | (98.9%) | 3 | (0.7%) | 2 | (0.5%) |  | 432 | (98.9%) | 4 | (0.9%) | 1 | (0.2%) |  | 0.788 |
| We have a reporting system (1st survey: “registration system”) for the use of anti-MRSA antibiotics. | 259 | (59.3%) | 173 | (39.6%) | 5 | (1.1%) |  | 425 | (97.3%) | 11 | (2.5%) | 1 | (0.2%) |  | **<.001** |
| **We have a preauthorization and/or restriction system for the use of anti-MRSA antibiotics.** | 206 | (47.1%) | 227 | (51.9%) | 4 | (0.9%) |  | 169 | (38.7%) | 265 | (60.6%) | 3 | (0.7%) |  | **0.035** |
| **We have criteria for the uses of broad-spectrum antibiotics such as carbapenems.** | 217 | (49.7%) | 216 | (49.4%) | 4 | (0.9%) |  | 224 | (51.3%) | 204 | (46.7%) | 9 | (2.1%) |  | 0.305 |
| We have a reporting system (1st survey: “registration system”) for the use of broad-spectrum antibiotics. | 251 | (57.4%) | 176 | (40.3%) | 10 | (2.3%) |  | 415 | (95.0%) | 19 | (4.3%) | 3 | (0.7%) |  | **<.001** |
| **We have a preauthorization and/or restriction system for the use of broad-spectrum antibiotics.** | 157 | (35.9%) | 274 | (62.7%) | 6 | (1.4%) |  | 111 | (25.4%) | 320 | (73.2%) | 6 | (1.4%) |  | **0.003** |
| We record the used amount of broad-spectrum antibiotics. | 429 | (98.2%) | 5 | (1.1%) | 3 | (0.7%) |  | 431 | (98.6%) | 3 | (0.7%) | 3 | (0.7%) |  | 0.777 |
| We have a reference system, such as the intranet of a booklet, for the antibiogram. | 371 | (84.9%) | 61 | (14.0%) | 5 | (1.1%) |  | 383 | (87.6%) | 50 | (11.4%) | 4 | (0.9%) |  | 0.499 |
| **We performed TDM** |  |  |  |  |  |  |  |  |  |  |  |  |  |  |  |
| basically all cases. | 273 | (62.5%) |  |  |  |  |  | 287 | (65.7%) |  |  |  |  |  | 0.193 |
| selected cases. | 154 | (35.2%) |  |  |  |  |  | 143 | (32.7%) |  |  |  |  |  |  |
| no cases. | 6 | (1.4%) |  |  |  |  |  | 1 | (0.2%) |  |  |  |  |  |  |
| (No response) | 4 | (0.9%) |  |  |  |  |  | 6 | (1.4%) |  |  |  |  |  |  |
| We record the vaccination proportion of employees who are HBsAg-negative. | 369 | (84.4%) | 57 | (13.0%) | 11 | (2.5%) |  | 378 | (86.5%) | 56 | (12.8%) | 3 | (0.7%) |  | 0.096 |
| We perform IGRAs for employees who are in contact with tuberculosis patients. | 404 | (92.4%) | 29 | (6.6%) | 4 | (0.9%) |  | 397 | (90.8%) | 37 | (8.5%) | 3 | (0.7%) |  | 0.556 |
| **We record employees’ immunization statuses for measles, rubella, chickenpox, and mumps (2nd survey: “for all of measles, rubella, chickenpox, and mumps”).** | 371 | (84.9%) | 57 | (13.0%) | 9 | (2.1%) |  | 273 | (62.5%) | 160 | (36.6%) | 4 | (0.9%) |  | **<.001** |
| We have a manual and a reporting system of needle punctures and sharp object injuries. | 437 | (100.0%) | 0 | (0.0%) | 0 | (0.0%) |  | 437 | (100.0%) | 0 | (0.0%) | 0 | (0.0%) |  | – |
| **Needle puncture and sharp object injuries are reported to a relevant department, such as ICT** |  |  |  |  |  |  |  |  |  |  |  |  |  |  |  |
| in approximately 100% of relevant cases. | 301 | (68.9%) |  |  |  |  |  | 307 | (70.3%) |  |  |  |  |  | 0.408 |
| in approximately 80% of relevant cases. | 92 | (21.1%) |  |  |  |  |  | 87 | (19.9%) |  |  |  |  |  |  |
| in approximately 50% of relevant cases. | 12 | (2.7%) |  |  |  |  |  | 19 | (4.3%) |  |  |  |  |  |  |
| in approximately 20% of relevant cases. | 15 | (3.4%) |  |  |  |  |  | 12 | (2.7%) |  |  |  |  |  |  |
| in approximately 0% of relevant cases. | 16 | (3.7%) |  |  |  |  |  | 9 | (2.1%) |  |  |  |  |  |  |
| (No response) | 1 | (0.2%) |  |  |  |  |  | 3 | (0.7%) |  |  |  |  |  |  |
| **ICT and/or ICPs check the number of isolated antimicrobial-resistant organisms and other microorganisms that are relevant to infection control** |  |  |  |  |  |  |  |  |  |  |  |  |  |  |  |
| on a daily basis. | 281 | (64.3%) |  |  |  |  |  | 286 | (65.4%) |  |  |  |  |  | 0.110 |
| regularly. | 147 | (33.6%) |  |  |  |  |  | 148 | (33.9%) |  |  |  |  |  |  |
| We do not have such activities. | 3 | (0.7%) |  |  |  |  |  | 3 | (0.7%) |  |  |  |  |  |  |
| (No response) | 6 | (1.4%) |  |  |  |  |  | 0 | (0.0%) |  |  |  |  |  |  |
| ICT and/or ICPs record the species and trends of isolated microorganisms on a type-of-sample and a ward-by-ward basis. | 413 | (94.5%) | 16 | (3.7%) | 8 | (1.8%) |  | 414 | (94.7%) | 20 | (4.6%) | 3 | (0.7%) |  | 0.257 |
| We have a direct and fast reporting system to the doctor-in-charge, such as e-mail and telephone, when microorganisms are isolated from a sample that is supposed to be aseptic (e.g., a blood sample). | 422 | (96.6%) | 13 | (3.0%) | 2 | (0.5%) |  | 431 | (98.6%) | 4 | (0.9%) | 2 | (0.5%) |  | 0.088 |
| **We perform surveillance for surgical site infections.** | 334 | (76.4%) | 103 | (23.6%) | 0 | (0.0%) |  | 355 | (81.2%) | 81 | (18.5%) | 1 | (0.2%) |  | 0.119 |
| **We perform surveillance for ventilator-associated pneumonia.** | 162 | (37.1%) | 272 | (62.2%) | 3 | (0.7%) |  | 175 | (40.0%) | 261 | (59.7%) | 1 | (0.2%) |  | 0.422 |
| **We perform surveillance for central line-associated bloodstream infections.** | 330 | (75.5%) | 102 | (23.3%) | 5 | (1.1%) |  | 351 | (80.3%) | 85 | (19.5%) | 1 | (0.2%) |  | 0.088 |
| **We perform surveillance for catheter-associated urinary tract infections.** | 224 | (51.3%) | 211 | (48.3%) | 2 | (0.5%) |  | 258 | (59.0%) | 178 | (40.7%) | 1 | (0.2%) |  | 0.063 |
| **We perform active surveillance cultures.** | 228 | (52.2%) | 208 | (47.6%) | 1 | (0.2%) |  | 219 | (50.1%) | 217 | (49.7%) | 1 | (0.2%) |  | 0.831 |
| We have an established manual for outbreaks. | 417 | (95.4%) | 10 | (2.3%) | 10 | (2.3%) |  | 419 | (95.9%) | 9 | (2.1%) | 9 | (2.1%) |  | 0.947 |
| **3. Preventive measures by the route of infections** |  |  |  |  |  |  |  |  |  |  |  |  |  |  |  |
| We have a manual for the outbreak of tuberculosis. | 435 | (99.5%) | 1 | (0.2%) | 1 | (0.2%) |  | 437 | (100.0%) | 0 | (0.0%) | 0 | (0.0%) |  | 0.368 |
| We have a manual for the outbreak of measles. | 398 | (91.1%) | 36 | (8.2%) | 3 | (0.7%) |  | 401 | (91.8%) | 36 | (8.2%) | 0 | (0.0%) |  | 0.222 |
| We have a manual for the outbreak of chickenpox. | 393 | (89.9%) | 41 | (9.4%) | 3 | (0.7%) |  | 395 | (90.4%) | 42 | (9.6%) | 0 | (0.0%) |  | 0.222 |
| We provide N95 masks at the outpatient emergency department and other outpatient departments. | 429 | (98.2%) | 7 | (1.6%) | 1 | (0.2%) |  | 432 | (98.9%) | 4 | (0.9%) | 1 | (0.2%) |  | 0.661 |
| We put a surgical mask on patients with suspected airborne infections while transporting. | 436 | (99.8%) | 1 | (0.2%) | 0 | (0.0%) |  | 436 | (99.8%) | 0 | (0.0%) | 1 | (0.2%) |  | 0.368 |
| Wearing an N95 mask is mandatory while entering the ward of a patient with suspected tuberculosis. | 436 | (99.8%) | 0 | (0.0%) | 1 | (0.2%) |  | 436 | (99.8%) | 0 | (0.0%) | 1 | (0.2%) |  | 1.000 |
| We have a manual for the outbreak of influenza. | 435 | (99.5%) | 1 | (0.2%) | 1 | (0.2%) |  | 435 | (99.5%) | 1 | (0.2%) | 1 | (0.2%) |  | 1.000 |
| Wearing a surgical mask while entering the ward of a patient with a droplet infection is instructed by a manual. | 432 | (98.9%) | 3 | (0.7%) | 2 | (0.5%) |  | 437 | (100.0%) | 0 | (0.0%) | 0 | (0.0%) |  | 0.081 |
| We provide surgical masks in the wards of patients with droplet infections. | 374 | (85.6%) | 61 | (14.0%) | 2 | (0.5%) |  | 380 | (87.0%) | 57 | (13.0%) | 0 | (0.0%) |  | 0.336 |
| We have a manual for cases in which MRSA is isolated from a patient. | 429 | (98.2%) | 7 | (1.6%) | 1 | (0.2%) |  | 433 | (99.1%) | 4 | (0.9%) | 0 | (0.0%) |  | 0.400 |
| Wearing disposable gloves and a gown is mandatory while entering the ward of a patient with suspected contagious diseases. | 399 | (91.3%) | 37 | (8.5%) | 1 | (0.2%) |  | 401 | (91.8%) | 34 | (7.8%) | 2 | (0.5%) |  | 0.793 |
| We provide alcohol-based hand sanitizers in all wards except for some special wards, such as the psychiatric ward. | 427 | (97.7%) | 9 | (2.1%) | 1 | (0.2%) |  | 428 | (97.9%) | 9 | (2.1%) | 0 | (0.0%) |  | 0.607 |
| We provide alcohol-based hand sanitizers in all outpatient departments. | 404 | (92.4%) | 32 | (7.3%) | 1 | (0.2%) |  | 415 | (95.0%) | 22 | (5.0%) | 0 | (0.0%) |  | 0.224 |
| **4. Maintenance of medical equipment** |  |  |  |  |  |  |  |  |  |  |  |  |  |  |  |
| We adopt closed urine drainage systems. | 419 | (95.9%) | 16 | (3.7%) | 2 | (0.5%) |  | 426 | (97.5%) | 10 | (2.3%) | 1 | (0.2%) |  | 0.412 |
| **We do not change catheters without blockages or infections regularly.** | 322 | (73.7%) | 112 | (25.6%) | 3 | (0.7%) |  | 323 | (73.9%) | 112 | (25.6%) | 2 | (0.5%) |  | 0.904 |
| We have a manual for the maintenance of ventilators. | 376 | (86.0%) | 53 | (12.1%) | 8 | (1.8%) |  | 388 | (88.8%) | 41 | (9.4%) | 8 | (1.8%) |  | 0.424 |
| We adopt closed tracheal suction systems. | 382 | (87.4%) | 52 | (11.9%) | 3 | (0.7%) |  | 381 | (87.2%) | 52 | (11.9%) | 4 | (0.9%) |  | 0.931 |
| We use sterile water for humidifiers. | 428 | (97.9%) | 7 | (1.6%) | 2 | (0.5%) |  | 426 | (97.5%) | 5 | (1.1%) | 6 | (1.4%) |  | 0.311 |
| **We perform regular oral cleansing for intubated patients in approximately 100% of relevant cases.** |  |  |  |  |  |  |  |  |  |  |  |  |  |  |  |
| in approximately 100% of relevant cases. | 340 | (77.8%) |  |  |  |  |  | 333 | (76.2%) |  |  |  |  |  | 0.226 |
| in approximately 80% of relevant cases. | 63 | (14.4%) |  |  |  |  |  | 72 | (16.5%) |  |  |  |  |  |  |
| in approximately 50% of relevant cases. | 9 | (2.1%) |  |  |  |  |  | 16 | (3.7%) |  |  |  |  |  |  |
| in approximately 20% of relevant cases. | 6 | (1.4%) |  |  |  |  |  | 4 | (0.9%) |  |  |  |  |  |  |
| in approximately 0% of relevant cases. | 17 | (3.9%) |  |  |  |  |  | 8 | (1.8%) |  |  |  |  |  |  |
| (No response) | 2 | (0.5%) |  |  |  |  |  | 4 | (0.9%) |  |  |  |  |  |  |
| We have a manual for the maintenance of central line catheters. | 418 | (95.7%) | 13 | (3.0%) | 6 | (1.4%) |  | 425 | (97.3%) | 10 | (2.3%) | 2 | (0.5%) |  | 0.294 |
| **We insert central line catheters under maximal barrier precautions** |  |  |  |  |  |  |  |  |  |  |  |  |  |  |  |
| in approximately 100% of relevant cases. | 163 | (37.3%) |  |  |  |  |  | 167 | (38.2%) |  |  |  |  |  | 0.150 |
| in approximately 80% of relevant cases. | 171 | (39.1%) |  |  |  |  |  | 190 | (43.5%) |  |  |  |  |  |  |
| in approximately 50% of relevant cases. | 57 | (13.0%) |  |  |  |  |  | 54 | (12.4%) |  |  |  |  |  |  |
| in approximately 20% of relevant cases. | 28 | (6.4%) |  |  |  |  |  | 16 | (3.7%) |  |  |  |  |  |  |
| in approximately 0% of relevant cases. | 16 | (3.7%) |  |  |  |  |  | 7 | (1.6%) |  |  |  |  |  |  |
| (No response) | 2 | (0.5%) |  |  |  |  |  | 3 | (0.7%) |  |  |  |  |  |  |
| **We prepare intravenous hyperalimentation admixtures on clean benches** |  |  |  |  |  |  |  |  |  |  |  |  |  |  |  |
| in approximately 100% of relevant cases. | 182 | (41.6%) |  |  |  |  |  | 175 | (40.0%) |  |  |  |  |  | 0.335 |
| in approximately 80% of relevant cases. | 111 | (25.4%) |  |  |  |  |  | 134 | (30.7%) |  |  |  |  |  |  |
| in approximately 50% of relevant cases. | 31 | (7.1%) |  |  |  |  |  | 27 | (6.2%) |  |  |  |  |  |  |
| in approximately 20% of relevant cases. | 40 | (9.2%) |  |  |  |  |  | 28 | (6.4%) |  |  |  |  |  |  |
| in approximately 0% of relevant cases. | 72 | (16.5%) |  |  |  |  |  | 70 | (16.0%) |  |  |  |  |  |  |
| (No response) | 1 | (0.2%) |  |  |  |  |  | 3 | (0.7%) |  |  |  |  |  |  |
| We use transparent dressings on the sites of catheter insertion to make them easy to inspect visually |  |  |  |  |  |  |  |  |  |  |  |  |  |  |  |
| in approximately 100% of relevant cases. | 357 | (81.7%) |  |  |  |  |  | 380 | (87.0%) |  |  |  |  |  | 0.112 |
| in approximately 80% of relevant cases. | 24 | (5.5%) |  |  |  |  |  | 43 | (9.8%) |  |  |  |  |  |  |
| in approximately 50% of relevant cases. | 0 | (0.0%) |  |  |  |  |  | 0 | (0.0%) |  |  |  |  |  |  |
| in approximately 20% of relevant cases. | 8 | (1.8%) |  |  |  |  |  | 5 | (1.1%) |  |  |  |  |  |  |
| in approximately 0% of relevant cases. | 18 | (4.1%) |  |  |  |  |  | 8 | (1.8%) |  |  |  |  |  |  |
| (No response) | 0 | (0.0%) |  |  |  |  |  | 1 | (0.2%) |  |  |  |  |  |  |
| **5. Standard precautions** |  |  |  |  |  |  |  |  |  |  |  |  |  |  |  |
| **We instruct new employees in hand hygiene by practical training sessions** | 229 | (52.4%) | 208 | (47.6%) | 0 | (0.0%) |  | 222 | (50.8%) | 214 | (49.0%) | 1 | (0.2%) |  |  |
| for all professions. | 229 | (52.4%) |  |  |  |  |  | 222 | (50.8%) |  |  |  |  |  | 0.700 |
| for selected professions. | 194 | (44.4%) |  |  |  |  |  | 202 | (46.2%) |  |  |  |  |  |  |
| We do not have such training. | 14 | (3.2%) |  |  |  |  |  | 12 | (2.7%) |  |  |  |  |  |  |
| (No response) | 0 | (0.0%) |  |  |  |  |  | 1 | (0.2%) |  |  |  |  |  |  |
| We evaluate the implementation of hand hygiene instructions of all wards at least once a year. | 389 | (89.0%) | 46 | (10.5%) | 2 | (0.5%) |  | 411 | (94.1%) | 25 | (5.7%) | 1 | (0.2%) |  | **0.028** |
| **We instruct new employees of all professions how to put on and remove PPE.** | 347 | (79.4%) | 90 | (20.6%) | 0 | (0.0%) |  | 330 | (75.5%) | 106 | (24.3%) | 1 | (0.2%) |  | 0.255 |
| **We instruct all employees in PPE by practical training sessions every year.** | 85 | (19.5%) | 352 | (80.5%) | 0 | (0.0%) |  | 80 | (18.3%) | 353 | (80.8%) | 4 | (0.9%) |  | 0.126 |
| **6. Wards** |  |  |  |  |  |  |  |  |  |  |  |  |  |  |  |
| We provide hand sanitizers at the entrance of all wards. | 426 | (97.5%) | 11 | (2.5%) | 0 | (0.0%) |  | 426 | (97.5%) | 10 | (2.3%) | 1 | (0.2%) |  | 0.593 |
| All medical devices (e.g., thermometers, stethoscopes) of single isolation rooms are patient-dedicated. | 423 | (96.8%) | 14 | (3.2%) | 0 | (0.0%) |  | 414 | (94.7%) | 21 | (4.8%) | 2 | (0.5%) |  | 0.174 |
| We check expiry dates of sterilized medical devices daily. | 415 | (95.0%) | 21 | (4.8%) | 1 | (0.2%) |  | 416 | (95.2%) | 20 | (4.6%) | 1 | (0.2%) |  | 0.987 |
| We check expiry dates of unused medications. | 429 | (98.2%) | 6 | (1.4%) | 2 | (0.5%) |  | 430 | (98.4%) | 4 | (0.9%) | 3 | (0.7%) |  | 0.741 |
| We have established guides for the expiry dates of opened medications. | 421 | (96.3%) | 15 | (3.4%) | 1 | (0.2%) |  | 422 | (96.6%) | 17 | (3.9%) | 3 | (0.7%) |  | 0.514 |
| All wards have at least one infection control link nurse. | 432 | (98.9%) | 4 | (0.9%) | 1 | (0.2%) |  | 429 | (98.2%) | 5 | (1.1%) | 3 | (0.7%) |  | 0.571 |
| **7. ICU** |  |  |  |  |  |  |  |  |  |  |  |  |  |  |  |
| **Medical professions do not change their shoes while entering ICU.** | 363 | (83.1%) | 1 | (0.2%) | 73 | (16.7%) |  | 335 | (76.7%) | 4 | (0.9%) | 98 | (22.4%) |  | **0.037** |
| **Medical professions are not recommended to wear gowns while entering ICU.** | 361 | (82.6%) | 2 | (0.5%) | 74 | (16.9%) |  | 337 | (77.1%) | 3 | (0.7%) | 97 | (22.2%) |  | 0.128 |
| **We have handwashing sinks at the entrance of ICU.** | 259 | (59.3%) | 105 | (24.0%) | 73 | (16.7%) |  | 248 | (56.8%) | 92 | (21.1%) | 97 | (22.2%) |  | 0.107 |
| **We provide hand sanitizers at the entrance of ICU.** | 362 | (82.8%) | 3 | (0.7%) | 72 | (16.5%) |  | 338 | (77.3%) | 2 | (0.5%) | 97 | (22.2%) |  | 0.095 |
| **We advise the patients’ families to use hand sanitizers or wash hands before and after entering ICU.** | 362 | (82.8%) | 3 | (0.7%) | 72 | (16.5%) |  | 339 | (77.6%) | 1 | (0.2%) | 97 | (22.2%) |  | 0.066 |
| **8. Operating room** |  |  |  |  |  |  |  |  |  |  |  |  |  |  |  |
| We do not change stretchers while entering operating rooms. | 334 | (76.4%) | 99 | (22.7%) | 4 | (0.9%) |  | 352 | (80.5%) | 79 | (18.1%) | 6 | (1.4%) |  | 0.211 |
| **Medical professions do not change their shoes while entering operating rooms.** | 263 | (60.2%) | 169 | (38.7%) | 5 | (1.1%) |  | 285 | (65.2%) | 147 | (33.6%) | 5 | (1.1%) |  | 0.299 |
| We do not provide sticky mats at the entrance of operation rooms. | 434 | (99.3%) | 2 | (0.5%) | 1 | (0.2%) |  | 432 | (98.9%) | 4 | (0.9%) | 1 | (0.2%) |  | 0.715 |
| We have established standards of surgical hand preparation. | 375 | (85.8%) | 59 | (13.5%) | 3 | (0.7%) |  | 381 | (87.2%) | 55 | (12.6%) | 1 | (0.2%) |  | 0.553 |
| We do not recommend the use of a brush for surgical hand preparation. | 419 | (95.9%) | 15 | (3.4%) | 3 | (0.7%) |  | 420 | (96.1%) | 13 | (3.0%) | 4 | (0.9%) |  | 0.867 |
| **9. Prevention of postoperative infections** |  |  |  |  |  |  |  |  |  |  |  |  |  |  |  |
| We use electric clippers or depilatory creams for patients who need to remove their hair before surgery in all departments. | 420 | (96.1%) | 13 | (3.0%) | 4 | (0.9%) |  | 418 | (95.7%) | 18 | (4.1%) | 1 | (0.2%) |  | 0.271 |
| We advise patients who can take a shower to take a shower on the night before or the morning of the day of surgery. | 410 | (93.8%) | 22 | (5.0%) | 5 | (1.1%) |  | 410 | (93.8%) | 25 | (5.7%) | 2 | (0.5%) |  | 0.478 |
| We recommend the administration of prophylactic antibiotics 30 minutes to 1 hour before the incision. | 421 | (96.3%) | 21 | (4.8%) | 4 | (0.9%) |  | 406 | (92.9%) | 26 | (5.9%) | 5 | (1.1%) |  | 0.710 |
| We have manuals to establish the duration of prophylactic antibiotics administration | 188 | (43.0%) | 242 | (55.4%) | 7 | (1.6%) |  | 214 | (49.0%) | 214 | (49.0%) | 9 | (2.1%) |  |  |
| in all departments. | 188 | (43.0%) |  |  |  |  |  | 214 | (49.0%) |  |  |  |  |  | 0.230 |
| in selected departments. | 137 | (31.4%) |  |  |  |  |  | 113 | (25.9%) |  |  |  |  |  |  |
| We do not have such manuals. | 105 | (24.0%) |  |  |  |  |  | 101 | (23.1%) |  |  |  |  |  |  |
| (No response) | 7 | (1.6%) |  |  |  |  |  | 9 | (2.1%) |  |  |  |  |  |  |
| **10. Management of food hygiene in hospitals** |  |  |  |  |  |  |  |  |  |  |  |  |  |  |  |
| We adopt dry kitchen systems for hospital meals. | 330 | (75.5%) | 81 | (18.5%) | 26 | (5.9%) |  | 356 | (81.5%) | 68 | (15.6%) | 13 | (3.0%) |  | **0.040** |
| **11. Management of medical waste** |  |  |  |  |  |  |  |  |  |  |  |  |  |  |  |
| We distinguish infectious waste from other waste and store it in a place inaccessible to non-authorized people. | 428 | (97.9%) | 9 | (2.1%) | 0 | (0.0%) |  | 427 | (97.7%) | 9 | (2.1%) | 1 | (0.2%) |  | 0.607 |
| **12. Cleaning, disinfection, and sterilization of instruments** |  |  |  |  |  |  |  |  |  |  |  |  |  |  |  |
| We do not pre-clean or pre-disinfect medical devices in wards. | 355 | (81.2%) | 80 | (18.3%) | 2 | (0.5%) |  | 368 | (84.2%) | 67 | (15.3%) | 2 | (0.5%) |  | 0.501 |
| We clean and disinfect endoscopes in accordance with the manuals or check them regularly. | 375 | (85.8%) | 60 | (13.7%) | 2 | (0.5%) |  | 372 | (85.1%) | 62 | (14.2%) | 3 | (0.7%) |  | 0.885 |

SD, standard deviation; IQR, interquartile range; ICD, infection control doctor; MD, medical doctor; PhD, doctor of philosophy; ICT, infection control team; JANIS, Japan Nosocomial Infections Surveillance; ICC, infection control committee; AST, antimicrobial stewardship team; MRSA, methicillin-resistant Staphylococcus aureus; TDM, therapeutic drug monitoring; HBsAg, hepatitis B surface antigen; IGRA, interferon-gamma release assay; ICP, infection control practitioner; PPE, personal protective equipment; ICU, intensive care unit.

Values are presented as medians (interquartile range) for numeric variables and numbers (%) for categorical variables.

Questions in bold indicate that the proportion of the most favorable answer was <80%.

*Student's t-test or Satterthwaite test as appropriate for continuous variables; Cochran-Mantel-Haenszel test for categorical variables.

P values in bold indicate P<.05.

# Table S2. Results of the 1st and 2nd questionnaire surveys (hospitals that responded to both surveys)

|  | 1st survey (n=437) | | | | | |  | 2nd survey (n=437) | | | | | |  |  |
| --- | --- | --- | --- | --- | --- | --- | --- | --- | --- | --- | --- | --- | --- | --- | --- |
| Question | Number of hospitals | Response rate | Mean | ±SD | Median | (IQR) |  | Number of hospitals | Response rate | Mean | ±SD | Median | (IQR) |  | *P** |
| Number of staff |  |  |  |  |  |  |  |  |  |  |  |  |  |  |  |
| Physician (full-time) | 414 | 94.7% | 124.4 | ±143.4 | 80 | (50–140) |  | 415 | 95.0% | 126.8 | ±127.3 | 81 | (50–137) |  | 0.805 |
| Infectious disease specialist | 327 | 74.8% | 0.8 | ±1.5 | 0 | (0–1) |  | 326 | 74.6% | 1.0 | ±2.3 | 0 | (0–1) |  | 0.300 |
| Infection control trainer | 318 | 72.8% | 0.5 | ±0.8 | 0 | (0–1) |  | 310 | 70.9% | 0.5 | ±1.0 | 0 | (0–1) |  | 0.258 |
| Certified microbiologist | 289 | 66.1% | 0.0 | ±0.3 | 0 | (0–0) |  | 285 | 65.2% | 0.1 | ±0.3 | 0 | (0–0) |  | 0.717 |
| Certified infection control doctor |  |  |  |  |  |  |  | 284 | 65.0% | 0.2 | ±1.6 | 0 | (0–0) |  |  |
| Nurse (full-time) | 405 | 92.7% | 427.3 | ±240.8 | 368 | (246–543) |  | 406 | 92.9% | 435.6 | ±250.3 | 371 | (251–561) |  | 0.629 |
| Certified nurse in infection control | 421 | 96.3% | 1.6 | ±0.9 | 2 | (1–2) |  | 420 | 96.1% | 1.7 | ±0.9 | 2 | (1–2) |  | 0.098 |
| Professional nurse for infection prevention and control | 316 | 72.3% | 0.2 | ±0.6 | 0 | (0–0) |  | 320 | 73.2% | 0.2 | ±0.8 | 0 | (0–0) |  | 0.496 |
| Certified nurse specialist in infection control nursing |  |  |  |  |  |  |  | 317 | 72.5% | 0.1 | ±0.3 | 0 | (0–0) |  |  |
| Master of infection control-related course | 311 | 71.2% | 0.1 | ±0.4 | 0 | (0–0) |  | 312 | 71.4% | 0.1 | ±0.3 | 0 | (0–0) |  | 0.190 |
| Laboratory technologist (full-time) | 420 | 96.1% | 30.3 | ±22.4 | 24 | (17–36.5) |  | 416 | 95.2% | 30.6 | ±21.2 | 24.5 | (17–37) |  | 0.819 |
| Certified laboratory technologist in infection control | 345 | 78.9% | 0.6 | ±0.9 | 0 | (0–1) |  | 346 | 79.2% | 0.6 | ±1.0 | 0 | (0–1) |  | 0.272 |
| Pharmacist (full-time) | 418 | 95.7% | 24.3 | ±16.9 | 19 | (14–28) |  | 417 | 95.4% | 25.4 | ±17.7 | 20 | (14–30) |  | 0.360 |
| Infection control pharmacy specialist | 329 | 75.3% | 0.3 | ±0.6 | 0 | (0–0) |  | 319 | 73.0% | 0.3 | ±0.5 | 0 | (0–0) |  | 0.660 |
| Certified pharmacist in infection control | 355 | 81.2% | 0.7 | ±0.8 | 1 | (0–1) |  | 356 | 81.5% | 0.8 | ±0.9 | 1 | (0–1) |  | 0.077 |
| Infectious disease chemotherapy pharmacist | 329 | 75.3% | 0.6 | ±1.0 | 0 | (0–1) |  | 337 | 77.1% | 0.7 | ±0.9 | 0 | (0–1) |  | 0.471 |
| Dietitian | 415 | 95.0% | 6.3 | ±3.7 | 5 | (4–8) |  | 404 | 92.4% | 6.6 | ±4.0 | 6 | (4–8) |  | 0.370 |
| Administrative staff | 408 | 93.4% | 69.7 | ±56.7 | 56 | (33–87) |  | 405 | 92.7% | 71.2 | ±60.6 | 56 | (33–89) |  | 0.718 |
| Certified ICD (MD or PhD) | 380 | 87.0% | 3.1 | ±2.4 | 3 | (2–4) |  | 377 | 86.3% | 3.3 | ±2.5 | 3 | (2–4) |  | 0.322 |
| Number of ICT member, crude | 422 | 96.6% | 12.8 | ±7.7 | 11 | (8–17) |  | 415 | 95.0% | 13.2 | ±8.4 | 11 | (7–17) |  | 0.530 |
| Physician | 430 | 98.4% | 3.6 | ±3.3 | 3 | (2–4) |  | 433 | 99.1% | 3.7 | ±3.7 | 3 | (2–4) |  | 0.576 |
| devoting ≥50% of working time to the ICT | 430 | 98.4% | 1.3 | ±1.3 | 1 | (0–2) |  | 433 | 99.1% | 1.3 | ±1.3 | 1 | (0–2) |  | 0.984 |
| devoting ≥80% of working time to the ICT | 430 | 98.4% | 0.1 | ±0.4 | 0 | (0–0) |  | 433 | 99.1% | 0.1 | ±0.6 | 0 | (0–0) |  | 0.648 |
| ≥3-years’ experience of infection control measures | 430 | 98.4% | 2.4 | ±1.9 | 2 | (1–3) |  | 433 | 99.1% | 2.4 | ±2.2 | 2 | (1–3) |  | 0.657 |
| Nurse | 428 | 97.9% | 3.4 | ±3.3 | 2 | (2–4) |  | 435 | 99.5% | 3.5 | ±4.3 | 2 | (2–4) |  | 0.757 |
| devoting ≥50% of working time to the ICT | 428 | 97.9% | 0.5 | ±1.0 | 0 | (0–1) |  | 435 | 99.5% | 0.5 | ±1.0 | 0 | (0–1) |  | 0.627 |
| devoting ≥80% of working time to the ICT | 428 | 97.9% | 1.0 | ±0.6 | 1 | (1–1) |  | 435 | 99.5% | 1.1 | ±0.6 | 1 | (1–1) |  | 0.265 |
| ≥5-years’ experience of infection control measures | 428 | 97.9% | 1.8 | ±1.4 | 2 | (1–2) |  | 435 | 99.5% | 1.8 | ±1.4 | 2 | (1–2) |  | 0.472 |
| Pharmacist | 428 | 97.9% | 1.9 | ±0.9 | 2 | (1–2) |  | 432 | 98.9% | 2.0 | ±1.0 | 2 | (1–2) |  | 0.242 |
| devoting ≥50% of working time to the ICT | 428 | 97.9% | 1.0 | ±0.9 | 1 | (0–1) |  | 432 | 98.9% | 1.0 | ±0.8 | 1 | (0–1) |  | 0.818 |
| devoting ≥80% of working time to the ICT | 428 | 97.9% | 0.1 | ±0.3 | 0 | (0–0) |  | 432 | 98.9% | 0.0 | ±0.3 | 0 | (0–0) |  | 0.409 |
| ≥3-years’ experience as a hospital pharmacist | 428 | 97.9% | 1.7 | ±1.2 | 2 | (1–2) |  | 437 | 100.0% | 1.6 | ±1.0 | 2 | (1–2) |  | 0.545 |
| Laboratory technologist | 429 | 98.2% | 1.8 | ±0.9 | 2 | (1–2) |  | 431 | 98.6% | 1.8 | ±1.0 | 2 | (1–2) |  | 0.709 |
| devoting ≥50% of working time to the ICT | 429 | 98.2% | 1.0 | ±0.9 | 1 | (0–1) |  | 431 | 98.6% | 1.0 | ±0.8 | 1 | (0–1) |  | 0.878 |
| devoting ≥80% of working time to the ICT | 429 | 98.2% | 0.1 | ±0.3 | 0 | (0–0) |  | 431 | 98.6% | 0.1 | ±0.5 | 0 | (0–0) |  | 0.941 |
| ≥3-years’ experience as a hospital technologist | 429 | 98.2% | 1.6 | ±1.4 | 1 | (1–2) |  | 437 | 100.0% | 1.6 | ±1.0 | 1 | (1–2) |  | 0.566 |
| Dietitian | 368 | 84.2% | 0.2 | ±0.5 | 0 | (0–0) |  | 347 | 79.4% | 0.2 | ±0.5 | 0 | (0–0) |  | 0.948 |
| devoting ≥50% of working time to the ICT | 368 | 84.2% | 0.1 | ±0.3 | 0 | (0–0) |  | 347 | 79.4% | 0.0 | ±0.2 | 0 | (0–0) |  | 0.213 |
| devoting ≥80% of working time to the ICT | 368 | 84.2% | 0.0 | ±0.1 | 0 | (0–0) |  | 347 | 79.4% | 0.0 | ±0.0 | 0 | (0–0) |  | 0.083 |
| Administrative staff | 402 | 92.0% | 1.2 | ±1.1 | 1 | (0–2) |  | 394 | 90.2% | 1.2 | ±1.0 | 1 | (1–2) |  | 0.926 |
| devoting ≥50% of working time to the ICT | 402 | 92.0% | 0.3 | ±0.6 | 0 | (0–0) |  | 394 | 90.2% | 0.3 | ±0.6 | 0 | (0–0) |  | 0.966 |
| devoting ≥80% of working time to the ICT | 402 | 92.0% | 0.2 | ±0.4 | 0 | (0–0) |  | 394 | 90.2% | 0.1 | ±0.4 | 0 | (0–0) |  | 0.441 |
| Full time equivalent, all | 422 | 62.2% | 3.2 | ±2.1 | 2.8 | (1.6–4.3) |  | 415 | 74.2% | 3.2 | ±2.1 | 2.8 | (1.8–4.1) |  | 0.920 |
| Physician | 430 | 63.4% | 0.7 | ±0.7 | 0.5 | (0–1) |  | 433 | 77.5% | 0.7 | ±0.8 | 0.5 | (0.5–1) |  | 0.830 |
| Nurse | 428 | 63.1% | 1.1 | ±0.6 | 0.8 | (0.8–1.3) |  | 435 | 77.8% | 1.1 | ±0.6 | 0.8 | (0.8–1.3) |  | 0.693 |
| Pharmacist | 428 | 63.1% | 0.5 | ±0.5 | 0.5 | (0–0.8) |  | 432 | 77.3% | 0.5 | ±0.5 | 0.5 | (0–0.65) |  | 0.531 |
| Laboratory technologist | 429 | 63.3% | 0.5 | ±0.5 | 0.5 | (0–1) |  | 431 | 77.1% | 0.5 | ±0.5 | 0.5 | (0–0.8) |  | 0.931 |
| Dietitian | 368 | 54.3% | 0.0 | ±0.2 | 0 | (0–0) |  | 347 | 62.1% | 0.0 | ±0.1 | 0 | (0–0) |  | 0.067 |
| Administrative staff | 402 | 59.3% | 0.3 | ±0.4 | 0 | (0–0.5) |  | 394 | 70.5% | 0.2 | ±0.4 | 0 | (0–0.5) |  | 0.524 |

|  | 1st survey (n=437) | | | | | |  | 2nd survey (n=437) | | | | | |  |  |
| --- | --- | --- | --- | --- | --- | --- | --- | --- | --- | --- | --- | --- | --- | --- | --- |
| Question | Yes | | No | | No response | |  | Yes | | No | | No response | |  | *P** |
| We performed bacterial culture, identification, and susceptibility tests basically in our hospital. | 355 | (81.2%) | 71 | (16.2%) | 11 | (2.5%) |  | 367 | (84.0%) | 64 | (14.6%) | 6 | (1.4%) |  | 0.362 |
| We have an active ICT. | 436 | (99.8%) | 1 | (0.2%) | 0 | (0.0%) |  | 435 | (99.5%) | 1 | (0.2%) | 1 | (0.2%) |  | 0.607 |
| We participate in JANIS programs. | 426 | (97.5%) | 9 | (2.1%) | 2 | (0.5%) |  | 432 | (98.9%) | 3 | (0.7%) | 2 | (0.5%) |  | 0.219 |
| Clinical laboratory division | 421 | (96.3%) |  |  |  |  |  | 422 | (96.6%) |  |  |  |  |  | 0.855 |
| Antimicrobial-resistant bacterial infection division | 228 | (52.2%) |  |  |  |  |  | 235 | (53.8%) |  |  |  |  |  | 0.635 |
| Surgical site infection division | 249 | (57.0%) |  |  |  |  |  | 259 | (59.3%) |  |  |  |  |  | 0.493 |
| Intensive care unit division | 80 | (18.3%) |  |  |  |  |  | 74 | (16.9%) |  |  |  |  |  | 0.595 |
| Neonatal intensive care unit division | 56 | (12.8%) |  |  |  |  |  | 51 | (11.7%) |  |  |  |  |  | 0.606 |
| **1. Organizational structure for nosocomial infection control** |  |  |  |  |  |  |  |  |  |  |  |  |  |  |  |
| The head of our hospital attends ICC almost every time. | 379 | (86.7%) | 50 | (11.4%) | 8 | (1.8%) |  | 369 | (84.4%) | 67 | (15.3%) | 1 | (0.2%) |  | **0.018** |
| We have a comprehensive hospital infection control manual that can be used all around our hospital. | 437 | (100.0%) | 0 | (0.0%) | 0 | (0.0%) |  | 437 | (100.0%) | 0 | (0.0%) | 0 | (0.0%) |  | – |
| We hold a workshop regarding countermeasures against hospital infection more than once a year. | 437 | (100.0%) | 0 | (0.0%) | 0 | (0.0%) |  | 437 | (100.0%) | 0 | (0.0%) | 0 | (0.0%) |  | – |
| We have tools, such as the intranet and bulletin boards, to inform our staff of hospital infection-related matters. | 434 | (99.3%) | 3 | (0.7%) | 0 | (0.0%) |  | 436 | (99.8%) | 1 | (0.2%) | 0 | (0.0%) |  | 0.317 |
| **2. Activities of ICT** |  |  |  |  |  |  |  |  |  |  |  |  |  |  |  |
| We hold a regular ICT meeting. | 410 | (93.8%) | 27 | (6.2%) | 0 | (0.0%) |  | 416 | (95.2%) | 20 | (4.6%) | 1 | (0.2%) |  | 0.353 |
| We provide consultation as an activity of the ICT. | 412 | (94.3%) | 25 | (5.7%) | 0 | (0.0%) |  | 407 | (93.1%) | 28 | (6.4%) | 2 | (0.5%) |  | 0.333 |
| **We have an AST (a member can work for both ICT and AST).** | 355 | (81.2%) | 78 | (17.8%) | 4 | (0.9%) |  | 305 | (69.8%) | 126 | (28.8%) | 6 | (1.4%) |  | **<.001** |
| We monitor the uses of antibiotics to assure their propriety. | 420 | (96.1%) | 13 | (3.0%) | 4 | (0.9%) |  | 431 | (98.6%) | 5 | (1.1%) | 1 | (0.2%) |  | 0.064 |
| We intervene to assure appropriate uses of antibiotics. | 410 | (93.8%) | 19 | (4.3%) | 8 | (1.8%) |  | 415 | (95.0%) | 19 | (4.3%) | 3 | (0.7%) |  | 0.317 |
| **We have established criteria of interventions, such as their administration duration and selection, for patients administered antibiotics.** | 304 | (69.6%) | 121 | (27.7%) | 12 | (2.7%) |  | 310 | (70.9%) | 112 | (25.6%) | 15 | (3.4%) |  | 0.691 |
| **We have criteria for the uses of anti-MRSA antibiotics.** | 267 | (61.1%) | 164 | (37.5%) | 6 | (1.4%) |  | 278 | (63.6%) | 151 | (34.6%) | 8 | (1.8%) |  | 0.594 |
| We record the used amount of anti-MRSA antibiotics. | 432 | (98.9%) | 3 | (0.7%) | 2 | (0.5%) |  | 432 | (98.9%) | 4 | (0.9%) | 1 | (0.2%) |  | 0.788 |
| We have a reporting system (1st survey: “registration system”) for the use of anti-MRSA antibiotics. | 259 | (59.3%) | 173 | (39.6%) | 5 | (1.1%) |  | 425 | (97.3%) | 11 | (2.5%) | 1 | (0.2%) |  | **<.001** |
| **We have a preauthorization and/or restriction system for the use of anti-MRSA antibiotics.** | 206 | (47.1%) | 227 | (51.9%) | 4 | (0.9%) |  | 169 | (38.7%) | 265 | (60.6%) | 3 | (0.7%) |  | **0.035** |
| **We have criteria for the uses of broad-spectrum antibiotics such as carbapenems.** | 217 | (49.7%) | 216 | (49.4%) | 4 | (0.9%) |  | 224 | (51.3%) | 204 | (46.7%) | 9 | (2.1%) |  | 0.305 |
| We have a reporting system (1st survey: “registration system”) for the use of broad-spectrum antibiotics. | 251 | (57.4%) | 176 | (40.3%) | 10 | (2.3%) |  | 415 | (95.0%) | 19 | (4.3%) | 3 | (0.7%) |  | **<.001** |
| **We have a preauthorization and/or restriction system for the use of broad-spectrum antibiotics.** | 157 | (35.9%) | 274 | (62.7%) | 6 | (1.4%) |  | 111 | (25.4%) | 320 | (73.2%) | 6 | (1.4%) |  | **0.003** |
| We record the used amount of broad-spectrum antibiotics. | 429 | (98.2%) | 5 | (1.1%) | 3 | (0.7%) |  | 431 | (98.6%) | 3 | (0.7%) | 3 | (0.7%) |  | 0.777 |
| We have a reference system, such as the intranet of a booklet, for the antibiogram. | 371 | (84.9%) | 61 | (14.0%) | 5 | (1.1%) |  | 383 | (87.6%) | 50 | (11.4%) | 4 | (0.9%) |  | 0.499 |
| **We performed TDM** |  |  |  |  |  |  |  |  |  |  |  |  |  |  |  |
| basically all cases. | 273 | (62.5%) |  |  |  |  |  | 287 | (65.7%) |  |  |  |  |  | 0.193 |
| selected cases. | 154 | (35.2%) |  |  |  |  |  | 143 | (32.7%) |  |  |  |  |  |  |
| no cases. | 6 | (1.4%) |  |  |  |  |  | 1 | (0.2%) |  |  |  |  |  |  |
| (No response) | 4 | (0.9%) |  |  |  |  |  | 6 | (1.4%) |  |  |  |  |  |  |
| We record the vaccination proportion of employees who are HBsAg-negative. | 369 | (84.4%) | 57 | (13.0%) | 11 | (2.5%) |  | 378 | (86.5%) | 56 | (12.8%) | 3 | (0.7%) |  | 0.096 |
| We perform IGRAs for employees who are in contact with tuberculosis patients. | 404 | (92.4%) | 29 | (6.6%) | 4 | (0.9%) |  | 397 | (90.8%) | 37 | (8.5%) | 3 | (0.7%) |  | 0.556 |
| **We record employees’ immunization statuses for measles, rubella, chickenpox, and mumps (2nd survey: “for all of measles, rubella, chickenpox, and mumps”).** | 371 | (84.9%) | 57 | (13.0%) | 9 | (2.1%) |  | 273 | (62.5%) | 160 | (36.6%) | 4 | (0.9%) |  | **<.001** |
| We have a manual and a reporting system of needle punctures and sharp object injuries. | 437 | (100.0%) | 0 | (0.0%) | 0 | (0.0%) |  | 437 | (100.0%) | 0 | (0.0%) | 0 | (0.0%) |  | – |
| **Needle puncture and sharp object injuries are reported to a relevant department, such as ICT** |  |  |  |  |  |  |  |  |  |  |  |  |  |  |  |
| in approximately 100% of relevant cases. | 301 | (68.9%) |  |  |  |  |  | 307 | (70.3%) |  |  |  |  |  | 0.408 |
| in approximately 80% of relevant cases. | 92 | (21.1%) |  |  |  |  |  | 87 | (19.9%) |  |  |  |  |  |  |
| in approximately 50% of relevant cases. | 12 | (2.7%) |  |  |  |  |  | 19 | (4.3%) |  |  |  |  |  |  |
| in approximately 20% of relevant cases. | 15 | (3.4%) |  |  |  |  |  | 12 | (2.7%) |  |  |  |  |  |  |
| in approximately 0% of relevant cases. | 16 | (3.7%) |  |  |  |  |  | 9 | (2.1%) |  |  |  |  |  |  |
| (No response) | 1 | (0.2%) |  |  |  |  |  | 3 | (0.7%) |  |  |  |  |  |  |
| **ICT and/or ICPs check the number of isolated antimicrobial-resistant organisms and other microorganisms that are relevant to infection control** |  |  |  |  |  |  |  |  |  |  |  |  |  |  |  |
| on a daily basis. | 281 | (64.3%) |  |  |  |  |  | 286 | (65.4%) |  |  |  |  |  | 0.110 |
| regularly. | 147 | (33.6%) |  |  |  |  |  | 148 | (33.9%) |  |  |  |  |  |  |
| We do not have such activities. | 3 | (0.7%) |  |  |  |  |  | 3 | (0.7%) |  |  |  |  |  |  |
| (No response) | 6 | (1.4%) |  |  |  |  |  | 0 | (0.0%) |  |  |  |  |  |  |
| ICT and/or ICPs record the species and trends of isolated microorganisms on a type-of-sample and a ward-by-ward basis. | 413 | (94.5%) | 16 | (3.7%) | 8 | (1.8%) |  | 414 | (94.7%) | 20 | (4.6%) | 3 | (0.7%) |  | 0.257 |
| We have a direct and fast reporting system to the doctor-in-charge, such as e-mail and telephone, when microorganisms are isolated from a sample that is supposed to be aseptic (e.g., a blood sample). | 422 | (96.6%) | 13 | (3.0%) | 2 | (0.5%) |  | 431 | (98.6%) | 4 | (0.9%) | 2 | (0.5%) |  | 0.088 |
| **We perform surveillance for surgical site infections.** | 334 | (76.4%) | 103 | (23.6%) | 0 | (0.0%) |  | 355 | (81.2%) | 81 | (18.5%) | 1 | (0.2%) |  | 0.119 |
| **We perform surveillance for ventilator-associated pneumonia.** | 162 | (37.1%) | 272 | (62.2%) | 3 | (0.7%) |  | 175 | (40.0%) | 261 | (59.7%) | 1 | (0.2%) |  | 0.422 |
| **We perform surveillance for central line-associated bloodstream infections.** | 330 | (75.5%) | 102 | (23.3%) | 5 | (1.1%) |  | 351 | (80.3%) | 85 | (19.5%) | 1 | (0.2%) |  | 0.088 |
| **We perform surveillance for catheter-associated urinary tract infections.** | 224 | (51.3%) | 211 | (48.3%) | 2 | (0.5%) |  | 258 | (59.0%) | 178 | (40.7%) | 1 | (0.2%) |  | 0.063 |
| **We perform active surveillance cultures.** | 228 | (52.2%) | 208 | (47.6%) | 1 | (0.2%) |  | 219 | (50.1%) | 217 | (49.7%) | 1 | (0.2%) |  | 0.831 |
| We have an established manual for outbreaks. | 417 | (95.4%) | 10 | (2.3%) | 10 | (2.3%) |  | 419 | (95.9%) | 9 | (2.1%) | 9 | (2.1%) |  | 0.947 |
| **3. Preventive measures by the route of infections** |  |  |  |  |  |  |  |  |  |  |  |  |  |  |  |
| We have a manual for the outbreak of tuberculosis. | 435 | (99.5%) | 1 | (0.2%) | 1 | (0.2%) |  | 437 | (100.0%) | 0 | (0.0%) | 0 | (0.0%) |  | 0.368 |
| We have a manual for the outbreak of measles. | 398 | (91.1%) | 36 | (8.2%) | 3 | (0.7%) |  | 401 | (91.8%) | 36 | (8.2%) | 0 | (0.0%) |  | 0.222 |
| We have a manual for the outbreak of chickenpox. | 393 | (89.9%) | 41 | (9.4%) | 3 | (0.7%) |  | 395 | (90.4%) | 42 | (9.6%) | 0 | (0.0%) |  | 0.222 |
| We provide N95 masks at the outpatient emergency department and other outpatient departments. | 429 | (98.2%) | 7 | (1.6%) | 1 | (0.2%) |  | 432 | (98.9%) | 4 | (0.9%) | 1 | (0.2%) |  | 0.661 |
| We put a surgical mask on patients with suspected airborne infections while transporting. | 436 | (99.8%) | 1 | (0.2%) | 0 | (0.0%) |  | 436 | (99.8%) | 0 | (0.0%) | 1 | (0.2%) |  | 0.368 |
| Wearing an N95 mask is mandatory while entering the ward of a patient with suspected tuberculosis. | 436 | (99.8%) | 0 | (0.0%) | 1 | (0.2%) |  | 436 | (99.8%) | 0 | (0.0%) | 1 | (0.2%) |  | 1.000 |
| We have a manual for the outbreak of influenza. | 435 | (99.5%) | 1 | (0.2%) | 1 | (0.2%) |  | 435 | (99.5%) | 1 | (0.2%) | 1 | (0.2%) |  | 1.000 |
| Wearing a surgical mask while entering the ward of a patient with a droplet infection is instructed by a manual. | 432 | (98.9%) | 3 | (0.7%) | 2 | (0.5%) |  | 437 | (100.0%) | 0 | (0.0%) | 0 | (0.0%) |  | 0.081 |
| We provide surgical masks in the wards of patients with droplet infections. | 374 | (85.6%) | 61 | (14.0%) | 2 | (0.5%) |  | 380 | (87.0%) | 57 | (13.0%) | 0 | (0.0%) |  | 0.336 |
| We have a manual for cases in which MRSA is isolated from a patient. | 429 | (98.2%) | 7 | (1.6%) | 1 | (0.2%) |  | 433 | (99.1%) | 4 | (0.9%) | 0 | (0.0%) |  | 0.400 |
| Wearing disposable gloves and a gown is mandatory while entering the ward of a patient with suspected contagious diseases. | 399 | (91.3%) | 37 | (8.5%) | 1 | (0.2%) |  | 401 | (91.8%) | 34 | (7.8%) | 2 | (0.5%) |  | 0.793 |
| We provide alcohol-based hand sanitizers in all wards except for some special wards, such as the psychiatric ward. | 427 | (97.7%) | 9 | (2.1%) | 1 | (0.2%) |  | 428 | (97.9%) | 9 | (2.1%) | 0 | (0.0%) |  | 0.607 |
| We provide alcohol-based hand sanitizers in all outpatient departments. | 404 | (92.4%) | 32 | (7.3%) | 1 | (0.2%) |  | 415 | (95.0%) | 22 | (5.0%) | 0 | (0.0%) |  | 0.224 |
| **4. Maintenance of medical equipment** |  |  |  |  |  |  |  |  |  |  |  |  |  |  |  |
| We adopt closed urine drainage systems. | 419 | (95.9%) | 16 | (3.7%) | 2 | (0.5%) |  | 426 | (97.5%) | 10 | (2.3%) | 1 | (0.2%) |  | 0.412 |
| **We do not change catheters without blockages or infections regularly.** | 322 | (73.7%) | 112 | (25.6%) | 3 | (0.7%) |  | 323 | (73.9%) | 112 | (25.6%) | 2 | (0.5%) |  | 0.904 |
| We have a manual for the maintenance of ventilators. | 376 | (86.0%) | 53 | (12.1%) | 8 | (1.8%) |  | 388 | (88.8%) | 41 | (9.4%) | 8 | (1.8%) |  | 0.424 |
| We adopt closed tracheal suction systems. | 382 | (87.4%) | 52 | (11.9%) | 3 | (0.7%) |  | 381 | (87.2%) | 52 | (11.9%) | 4 | (0.9%) |  | 0.931 |
| We use sterile water for humidifiers. | 428 | (97.9%) | 7 | (1.6%) | 2 | (0.5%) |  | 426 | (97.5%) | 5 | (1.1%) | 6 | (1.4%) |  | 0.311 |
| **We perform regular oral cleansing for intubated patients in approximately 100% of relevant cases.** |  |  |  |  |  |  |  |  |  |  |  |  |  |  |  |
| in approximately 100% of relevant cases. | 340 | (77.8%) |  |  |  |  |  | 333 | (76.2%) |  |  |  |  |  | 0.226 |
| in approximately 80% of relevant cases. | 63 | (14.4%) |  |  |  |  |  | 72 | (16.5%) |  |  |  |  |  |  |
| in approximately 50% of relevant cases. | 9 | (2.1%) |  |  |  |  |  | 16 | (3.7%) |  |  |  |  |  |  |
| in approximately 20% of relevant cases. | 6 | (1.4%) |  |  |  |  |  | 4 | (0.9%) |  |  |  |  |  |  |
| in approximately 0% of relevant cases. | 17 | (3.9%) |  |  |  |  |  | 8 | (1.8%) |  |  |  |  |  |  |
| (No response) | 2 | (0.5%) |  |  |  |  |  | 4 | (0.9%) |  |  |  |  |  |  |
| We have a manual for the maintenance of central line catheters. | 418 | (95.7%) | 13 | (3.0%) | 6 | (1.4%) |  | 425 | (97.3%) | 10 | (2.3%) | 2 | (0.5%) |  | 0.294 |
| **We insert central line catheters under maximal barrier precautions** |  |  |  |  |  |  |  |  |  |  |  |  |  |  |  |
| in approximately 100% of relevant cases. | 163 | (37.3%) |  |  |  |  |  | 167 | (38.2%) |  |  |  |  |  | 0.150 |
| in approximately 80% of relevant cases. | 171 | (39.1%) |  |  |  |  |  | 190 | (43.5%) |  |  |  |  |  |  |
| in approximately 50% of relevant cases. | 57 | (13.0%) |  |  |  |  |  | 54 | (12.4%) |  |  |  |  |  |  |
| in approximately 20% of relevant cases. | 28 | (6.4%) |  |  |  |  |  | 16 | (3.7%) |  |  |  |  |  |  |
| in approximately 0% of relevant cases. | 16 | (3.7%) |  |  |  |  |  | 7 | (1.6%) |  |  |  |  |  |  |
| (No response) | 2 | (0.5%) |  |  |  |  |  | 3 | (0.7%) |  |  |  |  |  |  |
| **We prepare intravenous hyperalimentation admixtures on clean benches** |  |  |  |  |  |  |  |  |  |  |  |  |  |  |  |
| in approximately 100% of relevant cases. | 182 | (41.6%) |  |  |  |  |  | 175 | (40.0%) |  |  |  |  |  | 0.335 |
| in approximately 80% of relevant cases. | 111 | (25.4%) |  |  |  |  |  | 134 | (30.7%) |  |  |  |  |  |  |
| in approximately 50% of relevant cases. | 31 | (7.1%) |  |  |  |  |  | 27 | (6.2%) |  |  |  |  |  |  |
| in approximately 20% of relevant cases. | 40 | (9.2%) |  |  |  |  |  | 28 | (6.4%) |  |  |  |  |  |  |
| in approximately 0% of relevant cases. | 72 | (16.5%) |  |  |  |  |  | 70 | (16.0%) |  |  |  |  |  |  |
| (No response) | 1 | (0.2%) |  |  |  |  |  | 3 | (0.7%) |  |  |  |  |  |  |
| We use transparent dressings on the sites of catheter insertion to make them easy to inspect visually |  |  |  |  |  |  |  |  |  |  |  |  |  |  |  |
| in approximately 100% of relevant cases. | 357 | (81.7%) |  |  |  |  |  | 380 | (87.0%) |  |  |  |  |  | 0.112 |
| in approximately 80% of relevant cases. | 24 | (5.5%) |  |  |  |  |  | 43 | (9.8%) |  |  |  |  |  |  |
| in approximately 50% of relevant cases. | 0 | (0.0%) |  |  |  |  |  | 0 | (0.0%) |  |  |  |  |  |  |
| in approximately 20% of relevant cases. | 8 | (1.8%) |  |  |  |  |  | 5 | (1.1%) |  |  |  |  |  |  |
| in approximately 0% of relevant cases. | 18 | (4.1%) |  |  |  |  |  | 8 | (1.8%) |  |  |  |  |  |  |
| (No response) | 0 | (0.0%) |  |  |  |  |  | 1 | (0.2%) |  |  |  |  |  |  |
| **5. Standard precautions** |  |  |  |  |  |  |  |  |  |  |  |  |  |  |  |
| **We instruct new employees in hand hygiene by practical training sessions** | 229 | (52.4%) | 208 | (47.6%) | 0 | (0.0%) |  | 222 | (50.8%) | 214 | (49.0%) | 1 | (0.2%) |  |  |
| for all professions. | 229 | (52.4%) |  |  |  |  |  | 222 | (50.8%) |  |  |  |  |  | 0.700 |
| for selected professions. | 194 | (44.4%) |  |  |  |  |  | 202 | (46.2%) |  |  |  |  |  |  |
| We do not have such training. | 14 | (3.2%) |  |  |  |  |  | 12 | (2.7%) |  |  |  |  |  |  |
| (No response) | 0 | (0.0%) |  |  |  |  |  | 1 | (0.2%) |  |  |  |  |  |  |
| We evaluate the implementation of hand hygiene instructions of all wards at least once a year. | 389 | (89.0%) | 46 | (10.5%) | 2 | (0.5%) |  | 411 | (94.1%) | 25 | (5.7%) | 1 | (0.2%) |  | **0.028** |
| **We instruct new employees of all professions how to put on and remove PPE.** | 347 | (79.4%) | 90 | (20.6%) | 0 | (0.0%) |  | 330 | (75.5%) | 106 | (24.3%) | 1 | (0.2%) |  | 0.255 |
| **We instruct all employees in PPE by practical training sessions every year.** | 85 | (19.5%) | 352 | (80.5%) | 0 | (0.0%) |  | 80 | (18.3%) | 353 | (80.8%) | 4 | (0.9%) |  | 0.126 |
| **6. Wards** |  |  |  |  |  |  |  |  |  |  |  |  |  |  |  |
| We provide hand sanitizers at the entrance of all wards. | 426 | (97.5%) | 11 | (2.5%) | 0 | (0.0%) |  | 426 | (97.5%) | 10 | (2.3%) | 1 | (0.2%) |  | 0.593 |
| All medical devices (e.g., thermometers, stethoscopes) of single isolation rooms are patient-dedicated. | 423 | (96.8%) | 14 | (3.2%) | 0 | (0.0%) |  | 414 | (94.7%) | 21 | (4.8%) | 2 | (0.5%) |  | 0.174 |
| We check expiry dates of sterilized medical devices daily. | 415 | (95.0%) | 21 | (4.8%) | 1 | (0.2%) |  | 416 | (95.2%) | 20 | (4.6%) | 1 | (0.2%) |  | 0.987 |
| We check expiry dates of unused medications. | 429 | (98.2%) | 6 | (1.4%) | 2 | (0.5%) |  | 430 | (98.4%) | 4 | (0.9%) | 3 | (0.7%) |  | 0.741 |
| We have established guides for the expiry dates of opened medications. | 421 | (96.3%) | 15 | (3.4%) | 1 | (0.2%) |  | 422 | (96.6%) | 17 | (3.9%) | 3 | (0.7%) |  | 0.514 |
| All wards have at least one infection control link nurse. | 432 | (98.9%) | 4 | (0.9%) | 1 | (0.2%) |  | 429 | (98.2%) | 5 | (1.1%) | 3 | (0.7%) |  | 0.571 |
| **7. ICU** |  |  |  |  |  |  |  |  |  |  |  |  |  |  |  |
| **Medical professions do not change their shoes while entering ICU.** | 363 | (83.1%) | 1 | (0.2%) | 73 | (16.7%) |  | 335 | (76.7%) | 4 | (0.9%) | 98 | (22.4%) |  | **0.037** |
| **Medical professions are not recommended to wear gowns while entering ICU.** | 361 | (82.6%) | 2 | (0.5%) | 74 | (16.9%) |  | 337 | (77.1%) | 3 | (0.7%) | 97 | (22.2%) |  | 0.128 |
| **We have handwashing sinks at the entrance of ICU.** | 259 | (59.3%) | 105 | (24.0%) | 73 | (16.7%) |  | 248 | (56.8%) | 92 | (21.1%) | 97 | (22.2%) |  | 0.107 |
| **We provide hand sanitizers at the entrance of ICU.** | 362 | (82.8%) | 3 | (0.7%) | 72 | (16.5%) |  | 338 | (77.3%) | 2 | (0.5%) | 97 | (22.2%) |  | 0.095 |
| **We advise the patients’ families to use hand sanitizers or wash hands before and after entering ICU.** | 362 | (82.8%) | 3 | (0.7%) | 72 | (16.5%) |  | 339 | (77.6%) | 1 | (0.2%) | 97 | (22.2%) |  | 0.066 |
| **8. Operating room** |  |  |  |  |  |  |  |  |  |  |  |  |  |  |  |
| We do not change stretchers while entering operating rooms. | 334 | (76.4%) | 99 | (22.7%) | 4 | (0.9%) |  | 352 | (80.5%) | 79 | (18.1%) | 6 | (1.4%) |  | 0.211 |
| **Medical professions do not change their shoes while entering operating rooms.** | 263 | (60.2%) | 169 | (38.7%) | 5 | (1.1%) |  | 285 | (65.2%) | 147 | (33.6%) | 5 | (1.1%) |  | 0.299 |
| We do not provide sticky mats at the entrance of operation rooms. | 434 | (99.3%) | 2 | (0.5%) | 1 | (0.2%) |  | 432 | (98.9%) | 4 | (0.9%) | 1 | (0.2%) |  | 0.715 |
| We have established standards of surgical hand preparation. | 375 | (85.8%) | 59 | (13.5%) | 3 | (0.7%) |  | 381 | (87.2%) | 55 | (12.6%) | 1 | (0.2%) |  | 0.553 |
| We do not recommend the use of a brush for surgical hand preparation. | 419 | (95.9%) | 15 | (3.4%) | 3 | (0.7%) |  | 420 | (96.1%) | 13 | (3.0%) | 4 | (0.9%) |  | 0.867 |
| **9. Prevention of postoperative infections** |  |  |  |  |  |  |  |  |  |  |  |  |  |  |  |
| We use electric clippers or depilatory creams for patients who need to remove their hair before surgery in all departments. | 420 | (96.1%) | 13 | (3.0%) | 4 | (0.9%) |  | 418 | (95.7%) | 18 | (4.1%) | 1 | (0.2%) |  | 0.271 |
| We advise patients who can take a shower to take a shower on the night before or the morning of the day of surgery. | 410 | (93.8%) | 22 | (5.0%) | 5 | (1.1%) |  | 410 | (93.8%) | 25 | (5.7%) | 2 | (0.5%) |  | 0.478 |
| We recommend the administration of prophylactic antibiotics 30 minutes to 1 hour before the incision. | 421 | (96.3%) | 21 | (4.8%) | 4 | (0.9%) |  | 406 | (92.9%) | 26 | (5.9%) | 5 | (1.1%) |  | 0.710 |
| We have manuals to establish the duration of prophylactic antibiotics administration | 188 | (43.0%) | 242 | (55.4%) | 7 | (1.6%) |  | 214 | (49.0%) | 214 | (49.0%) | 9 | (2.1%) |  |  |
| in all departments. | 188 | (43.0%) |  |  |  |  |  | 214 | (49.0%) |  |  |  |  |  | 0.230 |
| in selected departments. | 137 | (31.4%) |  |  |  |  |  | 113 | (25.9%) |  |  |  |  |  |  |
| We do not have such manuals. | 105 | (24.0%) |  |  |  |  |  | 101 | (23.1%) |  |  |  |  |  |  |
| (No response) | 7 | (1.6%) |  |  |  |  |  | 9 | (2.1%) |  |  |  |  |  |  |
| **10. Management of food hygiene in hospitals** |  |  |  |  |  |  |  |  |  |  |  |  |  |  |  |
| We adopt dry kitchen systems for hospital meals. | 330 | (75.5%) | 81 | (18.5%) | 26 | (5.9%) |  | 356 | (81.5%) | 68 | (15.6%) | 13 | (3.0%) |  | **0.040** |
| **11. Management of medical waste** |  |  |  |  |  |  |  |  |  |  |  |  |  |  |  |
| We distinguish infectious waste from other waste and store it in a place inaccessible to non-authorized people. | 428 | (97.9%) | 9 | (2.1%) | 0 | (0.0%) |  | 427 | (97.7%) | 9 | (2.1%) | 1 | (0.2%) |  | 0.607 |
| **12. Cleaning, disinfection, and sterilization of instruments** |  |  |  |  |  |  |  |  |  |  |  |  |  |  |  |
| We do not pre-clean or pre-disinfect medical devices in wards. | 355 | (81.2%) | 80 | (18.3%) | 2 | (0.5%) |  | 368 | (84.2%) | 67 | (15.3%) | 2 | (0.5%) |  | 0.501 |
| We clean and disinfect endoscopes in accordance with the manuals or check them regularly. | 375 | (85.8%) | 60 | (13.7%) | 2 | (0.5%) |  | 372 | (85.1%) | 62 | (14.2%) | 3 | (0.7%) |  | 0.885 |

SD, standard deviation; IQR, interquartile range; ICD, infection control doctor; MD, medical doctor; PhD, doctor of philosophy; ICT, infection control team; JANIS, Japan Nosocomial Infections Surveillance; ICC, infection control committee; AST, antimicrobial stewardship team; MRSA, methicillin-resistant Staphylococcus aureus; TDM, therapeutic drug monitoring; HBsAg, hepatitis B surface antigen; IGRA, interferon-gamma release assay; ICP, infection control practitioner; PPE, personal protective equipment; ICU, intensive care unit.

Values are presented as medians (interquartile range) for numeric variables and numbers (%) for categorical variables.

Questions in bold indicate that the proportion of the most favorable answer was <80%.

*Student's t-test or Satterthwaite test as appropriate for continuous variables; Cochran-Mantel-Haenszel test for categorical variables.

P values in bold indicate P<.05.

# Table S3. Fit statistics of the latent status analysis

| Number of Classes | Log-likelihood | G^2^ | AIC | BIC |
| --- | --- | --- | --- | --- |
| 2 | -11098.2 | 16882.56 | 16972.6 | 17156.2 |
| 3 | -10850.5 | 16387.14 | 16529.1 | 16818.8 |
| 4 | -10680.0 | 16046.22 | 16244.2 | 16648.1 |
| 5 | -10510.7 | 15707.52 | 15965.5 | 16491.8 |
| 6 | -10491.5 | 15669.14 | 15991.1 | 16648.0 |
| 7 | -10365.6 | 15417.24 | 15807.2 | 16602.8 |

G^2^, deviance statistic; AIC, Akaike Information Criterion; BIC, Schwarz Bayesian Information Criterion.

# Figure S1. Item-response probabilities for each question by identified statuses.


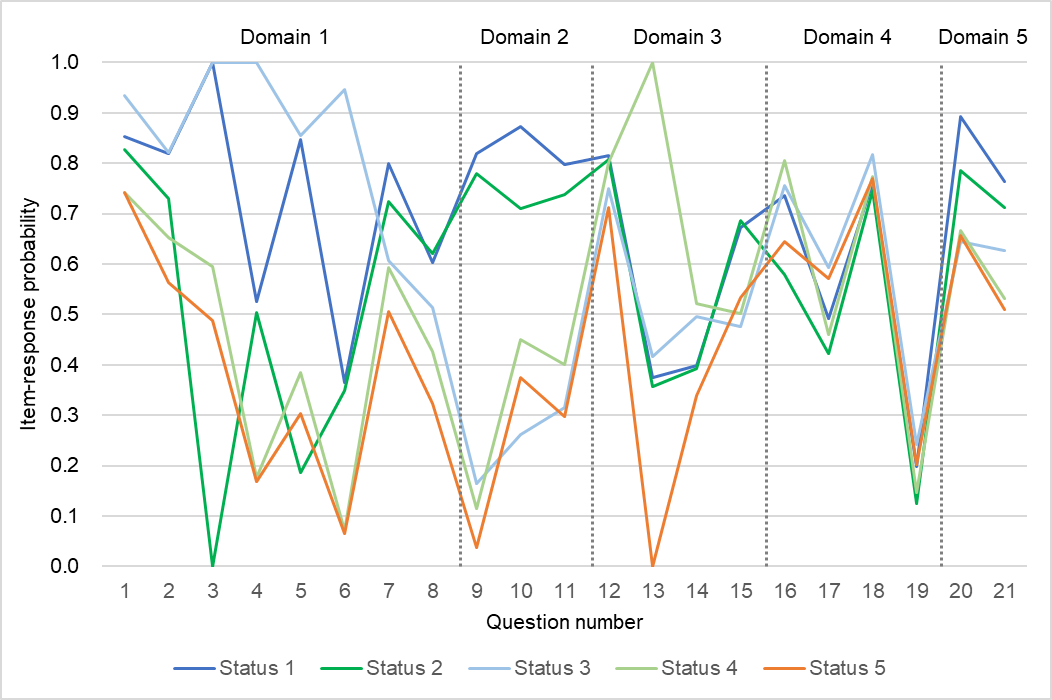


Domain 1, “antimicrobial stewardship”; domain 2, “surveillance”; domain 3, “medical and hospital equipment”; domain 4, “infection control team activities regarding vaccinations and education of employees”; domain 5, acknowledgment of updating relevant guidelines.”
